# Comparative evaluation of bulk and nano-formulated calcium, phosphorus, and potassium fertilizers in improving salinity tolerance of faba bean

**DOI:** 10.1186/s12870-026-09205-2

**Published:** 2026-06-15

**Authors:** Nesma M. Helal, Mervat S. Shamoon, Alyaa S. Abdel Halim, Khaled Yehia Farroh, Ibrahim M. El-Metwally, Hemmat I. Khattab, Heba M. Hassan

**Affiliations:** 1 Botany Department, Faculty of Science, Ain Shams University, Abbassia, Cairo Egypt; 2 Technology and Advanced Materials Central Lab, Agricultural Research Center, Giza, Egypt; 3 Biochemistry Department, Faculty of Science, Ain Shams University, P.O. 11566, Abbassia, Cairo Egypt; 4 Regional Center for Food and Feed, Agricultural Research Center, Giza, Egypt; 5 Botany Department, Agricultural and Biological Institute, National Research Center, Giza, Egypt

**Keywords:** Salinity stress, Faba bean, Photosynthetic pigments, Ion transporter genes, Antioxidant activity, Foliar application

## Abstract

**Background:**

Engineered nanoparticles (ENPs) have attracted considerable attention because of their potential role in enhancing crop tolerance to abiotic stresses, particularly salinity. Salinity is one of the major environmental constraints limiting crop productivity worldwide, as it adversely affects plant growth, physiological performance, nutrient uptake, and ionic balance.

**Results:**

In this context, the present study evaluated the effects of foliar application of calcium–phosphate–potassium (CaPK) fertilizers in both bulk and nanoparticle (CaPK-NP) forms on the growth, physiological performance, and gene expression analysis of faba bean (*Vicia faba* L.) under saline field conditions during two growing seasons (2021–2022). To achieve this objective, foliar sprays of bulk CaPK and CaPK-NPs were applied at concentrations of 8 and 16 mL L⁻¹. Key growth traits, photosynthetic performance, antioxidant activity, ion balance, and the expression of selected ion transporter genes namely *SOS1*,* VHA2*,* VFK1*, and *KUP7* were assessed to better understand the mechanisms underlying salt stress mitigation. The results showed that the 8 mL L⁻¹ bulk CaPK treatment consistently improved growth, photosynthetic pigments, antioxidant responses, nutrient uptake, and the Na⁺/K⁺ balance compared with untreated plants. Furthermore, gene expression analysis demonstrated the upregulation of ion transport-related genes, suggesting improved ionic homeostasis under saline conditions. Notably, the application of 16 mL L⁻¹ bulk CaPK resulted in the greatest enhancement of yield parameters. Although CaPK-NPs also improved several measured parameters, bulk CaPK frequently produced comparable or even greater enhancements under the experimental conditions.

**Conclusions:**

These findings suggest that foliar CaPK application may contribute to improved salinity tolerance in faba bean. However, further multi-location studies and long-term environmental assessments are still required before making broader agricultural recommendations.

**Supplementary Information:**

The online version contains supplementary material available at 10.1186/s12870-026-09205-2.

## Background

Abiotic environmental stresses such as drought, salinity, extreme heat or cold, and heavy metal contamination are key limiting factors of global crop productivity, negatively affecting plant physiology and yield by disrupting water relations, ion balance, and metabolic functions [1, 2]. Salinity stress, in particular, undermines plant growth and productivity by impairing nutrient uptake and causing ionic toxicity, making it a major challenge for sustainable agriculture. In Egypt, nearly one-third of cultivated land is affected by salinization, especially in dry and semi-arid regions. Salt-affected soil occupies about 1.38 billion hectares, or 10.7% of global land, with about 10% of both irrigated and rain-fed agricultural areas impacted by salinity [3]. Salinity causes yield losses reached about 20–70% in sensitive crops such as rice, wheat, and legumes. In arid and semi-arid regions, including Egypt, salinity-driven reductions pose an increasing threat to food security due to intensive irrigation and climate change [4]. Salt stress tolerance in plants is a complicated trait influenced by various genetic, physiological, and biochemical mechanisms. Elevated salt concentrations in soil impede the water absorption and transport of mineral nutrients, resulting in an increase of toxic ions in plants. This disrupts growth, destabilizes plasma membranes, induces reactive oxygen species (ROS) production, impairs metabolic processes like photosynthesis, and reduces enzymatic activity [5, 6]. Plant resistance to salt stress relies heavily on maintaining cellular ion homeostasis, facilitated by coordinated mechanisms including controlled ion absorption, vacuolar sequestration, and active ion extrusion [7, 8]. Plants evolved many strategies such as genetic engineering, modulation of metabolic pathways, and accumulation of osmoprotectants like glycine betaine and proline [9, 10] to enhance salt stress tolerance. These mechanisms help alleviate cellular water equilibrium and activate antioxidant defense systems that mitigate ROS damage under salt stress [7]. Additionally, the expression of key ion transporter genes including potassium transporters, sodium antiporters, and plasma membrane H⁺-ATPases plays a critical role in ionic homeostasis. Salt tolerance is also closely regulated by the Salt Overly Sensitive (SOS) signaling pathway and potassium transport systems such as HAK/KUP [11, 12]. Notably, efficient nutrient management is fundamental to sustainable crop production, especially under stress conditions such as salinity. Calcium, potassium, and phosphorus are vital macronutrients involved in crucial metabolic processes and significantly alleviate the detrimental impacts of salinity on crops such as rice and common bean [13, 14]. However, excessive application of conventional fertilizers containing these nutrients can lead to environmental problems, including micronutrient imbalances, groundwater contamination, leaching, and eutrophication [15]. To manage those obstacles and promote sustainable agricultural practices, there is an urgent need for novel fertilizer delivery technologies that improve efficiency while minimizing environmental impact. In plants, nanoparticles play a significant role in improving growth and stress tolerance by enhancing nutrient uptake, regulating ion homeostasis, and activating antioxidant defense systems [16, 17]. Under salt stress conditions, several studies have demonstrated that nanoparticles mitigate ionic and osmotic stress by reducing Na⁺ accumulation, alleviating oxidative damage through enhanced antioxidant enzyme activity, and improving photosynthetic efficiency [18, 19]. Consequently, the application of nanoparticles represents an innovative and effective strategy for enhancing crop productivity and resilience in saline environments [20] with reducing dependency on chemical fertilizers and minimizing environmental pollution [21]. These effects are often mediated by the upregulation of stress-responsive genes and proteins associated with key biochemical pathways.

Although engineered nanoparticles may raise concerns regarding environmental safety, the use of nutrient-based CaPK nanoparticles at low foliar doses, combined with their essential elemental composition and absence of phytotoxic effects, suggests that potential environmental risks under the conditions of this study are limited. Nevertheless, long-term field-scale assessments addressing nanoparticle persistence, soil interactions, and impacts on non-target organisms are still required before large-scale agricultural adoption.

Despite their benefits, ENPs such as cupper-oxide (CuO-NPs) and titanium-oxide (TiO₂-NPs) can pose toxicity risks to plants, animals, and humans if not properly managed. Therefore, extensive risk assessments and regulatory measures are imperative to guarantee the safe application of ENPs in agriculture. Faba bean (*Vicia faba* L.) is a vital leguminous crop with significant economic importance, particularly in Mediterranean regions like Egypt, where it is a staple food due to its high nutritional value [22]. Beyond its role as a food source, legumes such as faba beans are crucial for sustainable agriculture, providing proteins, carbohydrates, minerals, and vitamins. They are also used as animal fodder, further emphasizing the need to enhance their global production [23, 24]. Additionally, faba beans can contribute to land reclamation efforts by forming nitrogen-fixing nodules; however, it is one of the most sensitive plants to abiotic stress, especially soil salinity. Faba bean (*Vicia faba* L.) is particularly sensitive to salt stress during early vegetative and flowering stages, which can result in stunted growth, chlorosis, reduced pod set, and lower nitrogen fixation efficiency [24, 25]. Misr 3 is a widely cultivated faba bean cultivar in Egypt, valued for its yield potential and adaptability; however, its productivity is notably constrained under saline soil conditions. This moderate salt sensitivity makes it an appropriate cultivar for assessing the efficiency of bulk and nano CaPK fertilizers in improving salt stress tolerance [26]. The application of calcium in soil remediation, particularly for salinity issues, could be an effective strategy for both Egyptian and global agricultural systems. Consequently, this study’s main goal is to assess the prospective benefits of nanofertilizers, specifically calcium-potassium-phosphorous nanoparticles, in justifying the detrimental effects of salt stress on *Vicia faba* plants.

We hypothesized that foliar nano Ca–K formulations enhance salinity tolerance in faba bean by improving nutrient efficiency, ion homeostasis, and stress-responsive mechanisms. This study compares bulk and nano CaPK under saline field conditions, linking agronomic performance with physiological and molecular responses.

## Materials and methods

### Material

Seeds of faba beans (*Vicia faba*) cultivar (Misr 3) were obtained from Ministry of Agriculture, Department of Legumes Crops Research, and Egypt. Seeds with comparable size and color were carefully chosen.

Calcium, potassium, and phosphate (CaPK) were applied either as conventional bulk soil fertilizers or as calcium-potassium-phosphate nanoparticles (CaPK-NPs) via foliar spray.

### Preparation and characterization of calcium phosphate hydroxide/potassium nanocomposite

**Fig. 1:**
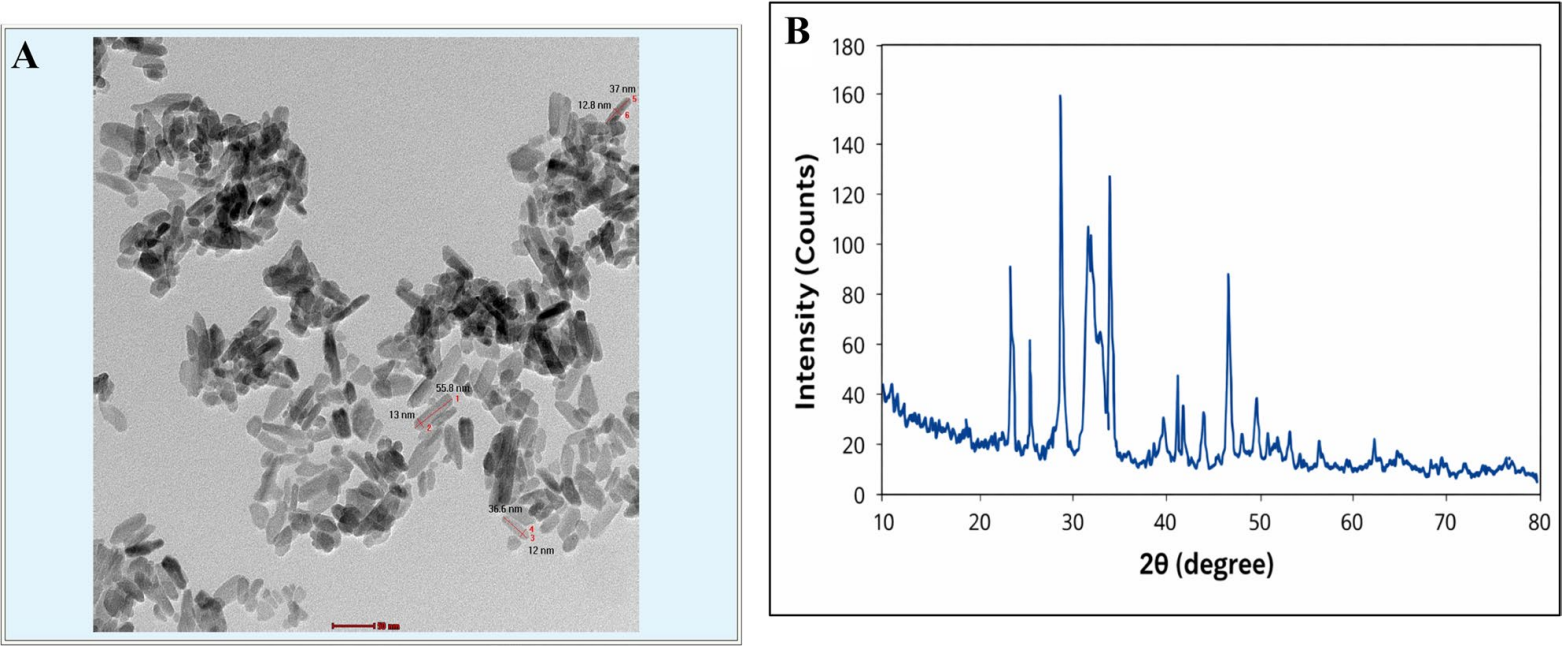
Characterization of CaPK NC. **A** HR-TEM image of CaPK NC. **B** XRD pattern analysis indicating the formation of CaPK NC

The synthesis of calcium phosphate hydroxide/potassium nanocomposite (CaPK NC) was conducted using the wet-chemical precipitation process subsequent an adapted version of the protocol described by Bianco et al. [27]. In the current work, Potassium nitrate (Assay ≥ 99.0%, % Sigma-Aldrich, St. Louis, MO, USA) was dissolved in deionized water (Milli-Q, Millipore, USA) and rapidly agitated at 1000 rpm/min for 30 min at 25 °C. After 12 h of vigorous stirring at 1000 rpm min^− 1^ with calcium hydroxide (Assay ≥ 95.0, %Sigma-Aldrich, St. Louis, MO, USA) added to the potassium nitrate solution, the resulting orthophosphoric acid (85%, %Sigma-Aldrich, St. Louis, MO, USA) solution was added dropwise. A molar ratio of Ca/*P* = 1.67 governs the reaction between the calcium and phosphate ions. The precipitated materials were allowed to settle for one evening prior to filtration, and the suspension was left to stand for 12 h to allow complete settling. An oven set to 100 °C was used to dry the filtered precipitate for two hours. A High-Resolution Transmission Electron Microscope (HR-TEM) running at an accelerating voltage of 200 kV (Tecnai G2, FEI, Netherlands) was used to image the real morphology of the as-prepared CaPK Nano Composite (NC) (Fig. 1). To lower particle aggregation, a diluted CaPK NC solution was ultrasonically sonicated for five minutes. Three drops of the sonicated solution were placed on a copper grid coated with carbon using a micropipette, and subsequently permitted to dry at ambient temperature. The CaPH NPs placed on the grid were imaged using high-resolution transmission electron microscopy (HR-TEM) to observe their morphology and size distribution. The X-ray diffraction (XRD) technique was employed to evaluate the chemical structure of CaPK NC as it was produced. The matching XRD pattern was obtained utilizing a Cu K radiation tube (= 1.54 Å) running at 40 kV and 30 mA in the scanning mode (X’pert PRO, PAN analytical, Netherlands). The standard ICCD library included with PDF4 software was used to interpret the acquired diffraction pattern. All steps of the preparation and characterization procedure were conducted at the Nanotechnology and Advanced Materials Central Lab (NAMCL), Agricultural Research Center, Egypt.

### Experimental site

Field experiments were executed in the winter seasons of 2020/2021 and 2021/2022 at the Research and Production Station of the National Research Centre (NRC) in El-Nubaria Province, El-Behira Governorate, Egypt (30.8667° N, 31.1667° E; 21 m a.s.l.). The site is categorized as arid to semi-arid. Meteorological data, including average minimum and maximum temperatures and relative humidity during both growing seasons, were collected from the weather station at the NRC, Nubaria, and are presented in Figure S1. Soil properties of the experimental site were analyzed following the method of Chapman and Pratt [28] and are shown in Table 1. Soil samples were extracted from a depth of 30 cm using an auger prior to faba bean planting for physico-chemical analysis. The soil texture and chemical properties of the experimental site are detailed in Table 1.

**Table 1:** Physical and chemical analyses of experimental soil

	pH	EC	CaCo_3_	OM%	Soluble Cations meq/l				Soluble anions meq/l			
	01:02.5	dSm^− 1^			Na^+^	K^+^	Mg^+^	Ca^++^	CO_3_^−−^	HCO_3_^−^	Cl^−^	SO_4_^−−^
Normal soil	7.7	0.11	2.8	0.26	0.08	0.02	0.1	0.25	0	0.1	1	0.07
Salt soil	8.18	1.03	2.3	0.9	5.1	0.7	1.3	3.1	0	1.4	7	1.8

### Experimental design

Uniform faba bean seeds were chosen based on size and color, surface-sterilized in 10% sodium hypochlorite for 15 min, thoroughly rinsed with distilled water, and air-dried. The field experiments were conducted during the 2020/2021 and 2021/2022 winter seasons. Seeds were sown at a rate of 7.00 kg ha⁻¹ on November 21 st in both seasons and harvested on May 22nd of the respective years. The experimental plots measured 3.5 × 3.0 m (10.5 m²), with 25 cm spacing between plots and 70 cm between ridges. Land preparation, then seed sowing, was performed. The experiment was arranged in a split-plot design, in which salinity levels were assigned to the main plots, while CaKP forms (bulk and nano) and their application rates were allocated to the subplots. This arrangement was adopted to efficiently evaluate the interaction between salinity stress and fertilizer treatments under field conditions. Thinning was done 21 days after sowing (DAS), leaving two plants per hill. The experiment employed a split-plot design with three replicates. Primary plots included two soil types (normal and saline), while sub-plots were assigned to the different treatments. Soil salinity was standardized prior to planting and maintained throughout the growing seasons. Soil electrical conductivity (EC) was periodically monitored during the experiment to ensure the stability of salinity levels across treatments. A preliminary germination test was conducted under various salinity levels (0.23, 1.03, 2.06, 3.09, 4.12, and 5.15 dS m⁻¹), and the most responsive concentration was selected based on plant growth performance.

### Fertilizers application

Foliar applications of calcium–potassium–phosphate in both bulk (CaPK) and nanoform (CaPK-NPs) were performed at concentrations of 8 and 16 mL L⁻¹ at 30 and 45 days after sowing (DAS). The application protocol followed the recommendations of the Nanotechnology and Advanced Material Central lab, Agricultural Research Center, Giza, Egypt. All treatments were administered via hand sprayer in the early morning (before 9:00 a.m.) at a fine droplet pressure. Approximately 500 L ha⁻¹ of solution was used, with plants sprayed from both sides to ensure full coverage. Control plants received distilled water.

### Irrigation and sampling

A drip irrigation system was employed, with irrigation intervals of every five days for two hours. After 75 days of sowing, leaf samples were collected for biochemical analysis. The experiment concluded at full maturity (182 DAS). From each plot, ten guarded plants were harvested to measure plant height, number of pods per plant, seeds per plant, pod weight per plant, 100-seed weight, and seed yield per plant (g). Total seed yield (kg acre⁻¹) was calculated based on the entire harvested plot. Seed yield quantity and quality were also assessed.

### Chemicals

All chemicals used in this study were of analytical reagent (AR) grade and were obtained from Sigma-Aldrich Chemical.

## Methods

### Photosynthetic pigments

Photosynthetic pigments were extracted from 1 g fresh leaf tissue using 85% aqueous acetone, and the filtrates were adjusted to 100 mL with the same solvent. Pigment concentrations including chlorophyll a, chlorophyll b, and carotenoids were quantified spectrophotometrically following the method of Metzner et al. [29]. Absorbance readings were recorded at 663, 644, and 452.5 nm against a blank of 85% acetone. Pigment concentrations (µg mL⁻¹) were calculated using specific equations based on the measured absorbance values.

$$\text{Chlorophyll a}=10.3\,\mathrm{E}_{663}-0.918\,\mathrm{E}_{644}$$

$$\text{Chlorophyll b}=19.7\,\mathrm{E}_{644}-3.870\,\mathrm{E}_{663}$$

$$\mathrm{Carotenoids}=4.2\,\mathrm{E}_{452.5}-\left(0.0264\, \text{chlorophyll a} +0.426\,\text{chlorophyll b}\right)$$

### Total soluble carbohydrates

Total soluble carbohydrates were extracted from 1 g of air-dried plant tissue using 10 mL of 80% (v/v) ethanol, following the procedure described by Homme et al. [30]. The mixture was heated in a boiling water bath to facilitate extraction and subsequently filtered. The filtrates were evaporated to dryness, and the residue was reconstituted in 10 mL of distilled water for subsequent analysis.

Soluble sugar content was determined according to the anthrone colorimetric method described by Blakeney and Mutton [31]. A known volume of plant extract or standard solution was mixed with 10 mL of freshly prepared anthrone reagent. The reaction mixture was heated in a boiling water bath for 15 min, then allowed to cool at room temperature. Absorbance was measured at 620 nm using a spectrophotometer, and carbohydrate concentration was calculated from a standard calibration curve.

### Total soluble proteins

Total soluble proteins were extracted by grinding in a solution of 0.1 M NaOH and 3.5% NaCl. and estimated according to Mæhre et al. [32]. The extraction was carried out in 3.5% NaCl. Then their concentration was determined by using Folin-Ciocalteu reagent. A volume of 0.1 mL of the plant protein extract was combined with 5 mL of alkaline reagent in a test tube. The solution was incubated at room temperature for at least 15 min. Then, 0.5 mL of diluted Folin-Ciocalteu reagent (1:2, v/v) was added and mixed immediately. After a 30-minute reaction period, absorbance was assessed at 700 nm utilizing a spectrophotometer.

### Proline

Proline content was determined utilizing the method of Bates et al. [33]. Fresh plant tissue (0.5 g) was homogenized in 10 mL of 3% sulfosalicylic acid and centrifuged at 12,000×g for 15 min. The supernatant was brought to a known final volume. A 2 mL aliquot of the supernatant was mixed with 2 mL of glacial acetic acid and ninhydrin reagent. The mixture was incubated in a boiling water bath for 1 h, then cooled. Subsequently, 4 mL of toluene were added, and the solution was vigorously shaken for 15–20 s. After phase separation in a separating funnel, the upper toluene layer was collected, and absorbance was measured at 520 nm using toluene as the blank.

### Lipid peroxidation product (MDA)

Malondialdehyde (MDA) level was quantified using the method of Minotti and Aust [34]. 100 mg of plant tissue was homogenized in 2 mL of distilled water and centrifuged at 4000 rpm for 15 min. An equal volume (2 mL) of 0.5% thiobarbituric acid (TBA) was mixed with the supernatant. The mixture was heated at 95 °C for 30 min, then cooled. Absorbance was recorded at 532 nm and 600 nm using a spectrophotometer, and the MDA content was calculated by subtracting the nonspecific absorbance at 600 nm from that at 532 nm.

### Total phenolic compounds

Total phenolic compounds were extracted and quantified following the method of Malik and Singh [35], with minor modifications. Total soluble phenols were extracted from 1 g fresh plant tissue with 80% (v/v) methanol at 0 °C. The extract was filtered through Whatman No. 1 filter paper, and the methanol was subsequently evaporated under reduced pressure. The dried residue was reconstituted to 10 mL with distilled water. For the estimation of total phenolics, 0.5 mL of the aqueous extract was mixed with 0.5 mL of Folin–Ciocalteu reagent and incubated for three minutes at room temperature. Subsequently, one mL of saturated sodium carbonate solution was added, and the mixture was allowed to react for one hour. The absorbance was measured at 725 nm using a spectrophotometer. Total phenolic content was calculated using a gallic acid calibration curve and expressed as milligrams of gallic acid equivalents per gram of sample.

### Hydrogen peroxide

Hydrogen peroxide (H_2_O_2_) levels were quantified according to the method described by Yu et al. [36]. Fresh plant tissue was homogenized in an ice bath using 0.1% (w/v) trichloroacetic acid (TCA). The homogenate was centrifuged at 12,000 × g for 15 min at 4 °C, and the resulting supernatant was collected. An aliquot of the supernatant was mixed with titanium reagent, which forms a yellow peroxytitanium complex in the presence of H_2_O_2_. The absorbance of this complex was measured at 415 nm using a UV–Visible spectrophotometer. The hydrogen peroxide concentration was calculated using a standard calibration curve and expressed as µmol H_2_O_2_ per gram fresh weight (FW).

### Antioxidant enzymes

Antioxidant enzyme activities were determined following the protocol of Mukherjee and Choudhuri [37]. Fresh plant tissue (2 g) was flash-frozen in liquid nitrogen and ground in a chilled mortar and pestle. The homogenate was extracted in 100 mM phosphate buffer (pH 6.8) and centrifuged at 20,000 × g for 20 min at 4 °C. The resulting supernatant was brought to a defined volume with the extraction buffer and used as the enzyme source. Superoxide dismutase (SOD) Activity was assayed according to Marklund and Marklund [38], based on the inhibition of pyrogallol auto-oxidation. The reaction mixture (10 mL) contained 3.6 mL distilled water, 0.1 mL enzyme extract, 5.5 mL of 50 mM phosphate buffer (pH 7.8), and 0.8 mL of 3 mM pyrogallol. The rate of pyrogallol oxidation was measured at 325 nm, and SOD activity was expressed in terms of inhibition percentage. Catalase (CAT) Activity was measured following Chen et al. [39]. An aliquot of enzyme extract (40 µL) was added to 9.96 mL of H₂O₂ solution. The decomposition rate of H_2_O_2_ was monitored by the decline in absorbance at 250 nm over 1 min, and CAT activity was calculated accordingly. Peroxidase (POX) Activity was estimated based on the method of Kar and Mishra [40]. The reaction mixture (5 ml) contained 300 µM phosphate buffer (pH 6.8), 50 µM catechol, 50 µM H_2_O_2_, and 1 mL of enzyme extract. After a 5-minute incubation at room temperature, the reaction was terminated with 1 mL of 10% H_2_SO_4_, and absorbance was recorded at 340 nm.

### Mineral elements

Mineral extraction was carried out following the method of Chapman and Pratt [41]. Finely ground plant material (2 g) was digested with 20 mL of a concentrated acid mixture (typically a combination of H_2_SO_4_ and HClO_4_) in a digestion flask under a fume hood. The mixture was initially heated at a low temperature, then gradually increased until dense sulphuric acid fumes were observed. After complete digestion, the solution was cooled, diluted to a known volume with distilled water, and stored in a stoppered flask for analysis.

Mineral concentrations were determined using a multi-parameter bench photometer. Nitrogen (N) content was measured using the Micro-Kjeldahl distillation method, as described by AOAC [42]. Sodium (Na) and potassium (K) were estimated according to the flame photometric method outlined by Page [43]. Phosphorus (P) content was quantified using the vanadomolybdate colorimetric method [44]. Calcium (Ca) was measured using the appropriate photometric settings on the instrument, following standard protocols.

### Gene expression analysis by quantitative real-time PCR (qPCR)

Gene expression levels of *VfSOS1*,* VfKUP7*,* VfVHA2*, and *VfVFK1* were determined in tissues using quantitative real-time polymerase chain reaction (qPCR). The primer sequences used for qPCRs were chosen based on information reported by Sagervanshi et al. [45], as shown in Table S1. Thermo Scientific (Waltham, Massachusetts, USA) was used for quantitative real-time analysis. *Vicia faba* cyclophilin (VfCYP) served as the endogenous control. Total RNA was extracted from each sample utilizing the Thermo Scientific Gene JET Plant RNA Purification Mini Kit (#K0801, Thermo Scientific, Waltham, MA, USA) according to the manufacturer’s instructions. DNase I was used to eliminate genomic DNA contamination from the RNA sample. The Revert Aid First Strand cDNA Synthesis Kit (#K1622, Thermo Scientific, Waltham, MA, USA) was used to generate first-strand cDNA from total RNA (1 µg). The Quant Inova SYBR Green PCR kit (Qiagen, Valencia, CA) was used according to the manufacturer’s instructions to quantify gene expression levels. The Quant Studio 3 Real-Time PCR System (Thermo Scientific, Waltham, Massachusetts, USA) was employed for quantitative real-time analysis. Each qPCR reaction was performed in triplicate three technical replicates, and the mean Ct value was used for subsequent expression analysis using the comparative Ct method (2^−ΔΔCt^) [46].

It should be noted that in the present study, primer amplification efficiencies for the target and reference genes were not experimentally validated by standard curve analysis prior to relative quantification analysis using the (2^−ΔΔCt^) method; therefore, this limitation may influence the absolute quantitative accuracy of gene expression estimates and should be considered when interpreting qPCR results.

Analyses of variance (ANOVA) for all data were calculated using SPSS v20.0 (SPSS Inc., Chicago, USA) analyzing software. Statistical significances of the means were compared with Duncan’s test at *p* ≤ 0.05 levels [47], the standard error (SE) of the means presented in tables, and the figures are means ± SE (number of replicates = 3).

## Results

### Characterization of calcium phosphate hydroxide/potassium nanocomposite

The physicochemical characterization of the synthesized CaPK nanocomposite (NC) is presented in Fig. 1. The characterization of the CaPK nanocomposite was performed by using different techniques. High-resolution transmission electron microscopy (HR-TEM) was employed to ascertain the particle shape and size. The HR-TEM image of the CaPK NC, shown in Fig. 1A, revealed that the particles exhibited a rod-like morphology, with a width of less than 12–13 nm and a length of approximately 37–56 nm. The phase formation of CaPK NC was further analyzed using X-ray diffraction (XRD), as depicted in Fig. 1B, established by Bragg’s reflections. The XRD pattern displayed sharp, intense, and narrow peaks at 25.84°, 31.8°, 32.18°, 32.95°, 39.86°, 46.73°, and 49.47°, corresponding to 2θ angles of (002), (211), (112), (300), (130), (222), and (213) hkl values. These peaks are attributed to the calcium phosphate hydroxide mixed with potassium, with the diffraction pattern aligning with the standard ICCD library (card no: 04–007−2837). Additionally, characteristic peaks at 23.55°, 23.82°, 29.41°, 32.95°, and 33.84° were observed, corresponding to the (111), (021), (012), and (112) planes of potassium, matching the standard potassium diffraction pattern (card no: 01–071−1558). The alignment of the diffraction peaks and their relative intensities confirmed that the synthesized nanoparticles were CaPK NC, consistent with the crystalline structure of calcium phosphate potassium nanocomposites.

### Effect of bulk CaPK and CaPKNPs on the yield components of the faba bean plant

This study evaluated the impact of different concentrations of calcium–potassium-based nanoparticles (CaPK-NPs) applied as foliar sprays on the growth, physiological, and biochemical responses of faba bean (*Vicia faba* L. cv. Misr 3) under salt stress conditions. The foliar application of either bulk or nano fertilizers verified positive improving effects on the growth and productivity of plants under normal and stressed conditions. According to the current findings, it was observed that plant height, stem weight, leaf weight, weight of pods per plant, number of pods per plant, weight of seed per plant, 100-seed weight, and seed yield ton per hectare of bean plants grown in saline soil were markedly reduced (Tables 2 and 3). However, foliar application of CaPK bulk fertilizer at 8 mL L^− 1^ and 16 mL L^−1^levels induced a noticeable significant increase in the previous parameters of unstressed and stressed faba bean plants (Tables 2 and 3). Notably, the percentage of increase in plant height reached about 108% and 106.3% after 90 days of sowing and the harvested stage, respectively, over the value of stressed faba bean plants (Table 2). Moreover, the maximum number of pods/plant (20.9 from 17.1) and weight of pods/plant (186.0 from 151.7), as well as weight of seed/plant (125.0 from 103.4), were observed in stressed plants treated with the high doses of bulk CaPK fertilizer (16 mL L^− 1^) as compared to those of the corresponding controls.

(Table 3). High seed yield ton ha^− 1^, 100-seed weight, and stem weight, as well as leaf weight, were recorded in unstressed and stressed plants at 16 mL L^− 1^, followed by 8 mL L^− 1^ bulk CaPK application (Table 3).

**Table 2:** Impact of CaPK nanoparticles and/or bulk material on some yield components of faba bean plants grown in sandy soil. Results are shown as a mean of 3 replicates ± SE. The values with same letter in the same column are non-significant and the value with different letters is significant

Criteria	Plant height at 90 days	Plant height at harvest	Stem weight(g)	Leaf weight(g)
Treatments				
Control (normal soil)	106.95 ± 1.21^b^	121.86 ± 1.35^b^	213.29 ± 4.89^b^	127.62 ± 4.62^d^
Salt Stress (sandy soil)	100 ± 1.78^d^	110.14 ± 0.92^e^	199.71 ± 4.9^d^	123.62 ± 4.03^e^
8 mL L^− 1^ NPs	105 ± 1.15^c^	116 ± 3.21^d^	212 ± 4.3^c^	131 ± 1.41^c^
16 mL L^− 1^ NPs	107.5 ± 2.32^b^	118 ± 3.21^c^	222 ± 3.62^a^	141.17 ± 2.09^a^
8 mL L^− 1^ NPs + salt	103 ± 1.15^c^	109 ± 1.15^f^	203 ± 1.73^d^	129 ± 1.73^c^
16 mL L^− 1^ NPs + salt	103 ± 1.15^c^	111 ± 1.15^e^	215 ± 2.89^b^	137.33 ± 2.03^b^
8 mL L^− 1^ CaPK	107.5 ± 0.99^b^	119.5 ± 3^b^	220 ± 2.89^a^	137.5 ± 1.57^b^
16 mL L^− 1^ CaPK	111 ± 1.53^a^	123 ± 2.78^a^	228 ± 2.49^a^	147.5 ± 2.22^a^
8 mL L^− 1^ CaPK + salt	106 ± 1.15^b^	113 ± 1.15^d^	215 ± 2.89^b^	135 ± 1.73^b^
16 mL L^− 1^ CaPK +salt	108 ± 1.15^b^	117 ± 1.15^c^	223 ± 1.73^a^	143 ± 1.73^a^

**Table 3:** Impact of CaPK nanoparticles and/or bulk material on some yield components of faba bean plants grown in sandy soil. Results are shown as a mean of 3 replicates ± SE. The values with same letter in the same column are non-significant and the value with different letters is significant

Criteria	Weight of pods/plant	Number of pods/plant	Weight of seed/plant	100 – seed weight	Seed yield ton/ha
Treatments					
Control (normal soil)	168.86 ± 5.73^c^	18.43 ± 0.66^c^	119.43 ± 3.42^d^	75.87 ± 0.84^c^	4.16 ± 0.08^b^
Salt Stress (sandy soil)	151.71 ± 5.13^e^	17.17 ± 0.78^d^	103.43 ± 3.89^f^	72.4 ± 1.2^e^	3.77 ± 0.09^e^
8 mL L^− 1^ NPs	159 ± 3.08^d^	17.65 ± 0.42^d^	109 ± 3.34^e^	73 ± 1.49^d^	4.1 ± 0.09^c^
16 mL L^− 1^ NPs	182 ± 4.94^b^	20.9 ± 0.45^b^	123.5 ± 1.73^b^	75.2 ± 1.15^c^	4.25 ± 0.08^b^
8 mL L^− 1^ NPs + salt	153 ± 1.73^e^	17.2 ± 0.58^d^	102 ± 1.15^f^	70.1 ± 1.15^f^	3.95 ± 0.12^e^
16 mL L^− 1^ NPs + salt	171 ± 0.52^c^	20.3 ± 0.58^b^	120 ± 1.15^c^	73.2 ± 1.15^d^	4.11 ± 0.06^c^
8 mL L^− 1^ CaPK	174.5 ± 5.2^b^	20.5 ± 0.69^b^	121 ± 3.8^b^	78.15 ± 0.89^b^	4.22 ± 0.11^b^
16 mL L^− 1^ CaPK	193 ± 3.7^a^	23.45 ± 0.37^a^	133.5 ± 4.0^a^	80.63 ± 0.67^a^	4.33 ± 0.09^a^
8 mL L^− 1^ CaPK + salt	163 ± 1.73^d^	19.2 ± 0.58^c^	113 ± 1.73^d^	77 ± 1.15^b^	4.0 ± 0.06^d^
16 mL L^− 1^ CaPK +salt	186 ± 3.46^a^	23.5 ± 0.58^a^	125 ± 2.89^a^	80.3 ± 1.15^a^	4.15 ± 0.09^b^

### Effect of bulk CaPK and CaPK NPs on the photosynthetic pigments of salt-stressed bean plant

In faba bean (cv. Misr 3), salt condition initiated a substantial decrease in chlorophylls a and b while simultaneously resulting in a pronounced increase in carotenoid level, which increased by 107.4% relative to control plants in salt-free soil (Table 4). The most notable enhancements were recorded at the 8 mL L^− 1^ bulk CaPK fertilizer, which increased chlorophylls a and b by 110.8% and 106.4%, and carotenoids by 104.9% above the levels of stressed plants (Table 4). Similarly, CaPK nanofertilizers further enhanced photosynthetic pigments, particularly at the 8 mL L^− 1^ nanofertilizer, where chlorophylls a and b increased by 115.4% and 116.5%, respectively, in unstressed plants. Conversely, higher doses of CaPK nano fertilizers led to reductions in photosynthetic pigments in both stressed and unstressed plants (Table 4).

**Table 4:** Impact of CaPK nanoparticles and/or bulk material on photosynthetic pigments of faba bean plants grown in sandy soil. Results are shown as a mean of 3 replicates ± SE. The values with same letter in the same column are non-significant and the value with different letters is significant

Parameters	Chlorophyll a (µg g^− 1^ FW)	Chlorophyll b (µg g^− 1^ FW)	Chl a/b	Total chlorophylls	Carotenoids (µg g^− 1^ FW)
Treatments					
Control (normal soil)	914.2 ± 8.19^a^	656.5 ± 4.8^a^	1.39 ± 0.0^c^	1888.55 ± 15.79^a^	317.86 ± 3.17^h^
Salt Stress (sandy soil)	723.99 ± 14.58^g^	509.57 ± 5.95^i^	1.42 ± 0.02^b^	1575.2 ± 22.25^i^	341.63 ± 2.29^d^
8 mL L^− 1^ NPs	835.68 ± 49.2^d^	593.91 ± 31.28^d^	1.4 ± 0.01^c^	1763.09 ± 77^d^	333.5 ± 3.49^f^
16 mL L^− 1^ NPs	818.53 ± 51.88^d^	584.92 ± 35.02^e^	1.4 ± 0.01^c^	1730.06 ± 82.45^e^	326.62 ± 4.49^g^
8 mL L^− 1^ NPs + salt	725.68 ± 1.15^g^	523.98 ± 1.41^h^	1.38 ± 0.01^c^	1590.91 ± 0.89^h^	341.25 ± 0.64^d^
16 mL L^− 1^ NPs + salt	702.55 ± 1.05^h^	506.65 ± 1.15^i^	1.39 ± 0.01^c^	1545.76 ± 0.73^j^	336.57 ± 0.63^e^
8 mL L^− 1^ CaPK	874.62 ± 32.18^b^	613.4 ± 31.86^b^	1.43 ± 0.02^b^	1834.98 ± 58.92^b^	346.96 ± 5.14^b^
16 mL L^− 1^ CaPK	863.57 ± 32.5^c^	605.65 ± 32.03^c^	1.43 ± 0.02^b^	1803.45 ± 59.68^c^	334.23 ± 4.89^f^
8 mL L^− 1^ CaPK + salt	802.65 ± 0.0^e^	542.2 ± 0.55^f^	1.48 ± 0.0^a^	1703.27 ± 0.16^f^	358.42 ± 0.71^a^
16 mL L^− 1^ CaPK +salt	790.9 ± 0.72^f^	534.05 ± 0.37^g^	1.48 ± 0.0^a^	1670.01 ± 1.58^g^	345.07 ± 1.22^c^

**Fig. 2:**
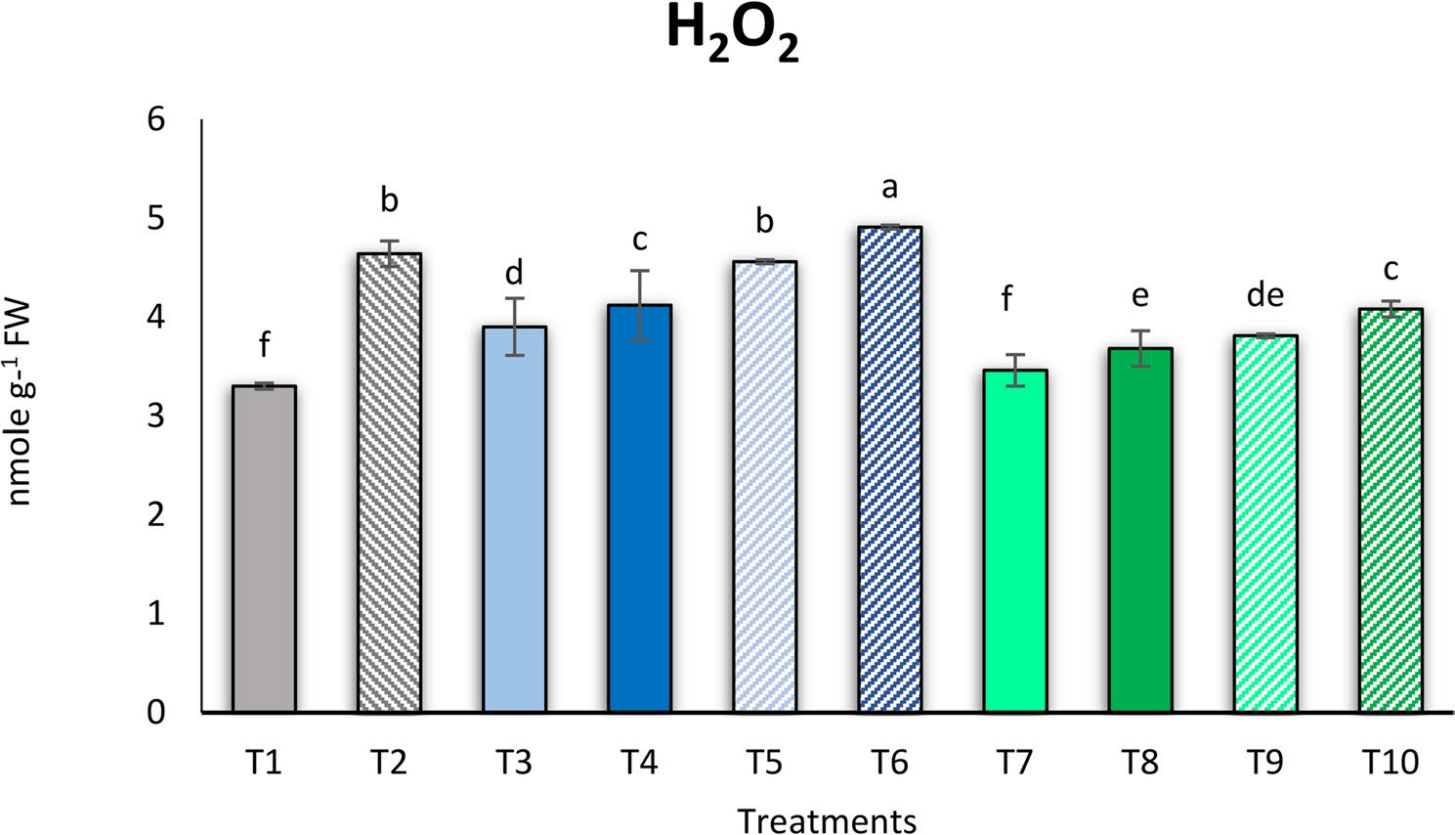
Impact of CaPK nanoparticles and/or bulk material on malondialdehyde (MDA) and hydrogen peroxide (H_2_O_2_) contents of faba bean plants grown in sandy soil. T1: control (normal soil), T2: salt stress (sandy soil), T3: 8 mL L^− 1^ NPs, T4: 16 mL L^− 1^ NPs, T5: 8 mL L^− 1^ NPs+ salt, T6: 16 mL L^− 1^ NPs+ salt, T7: 8 mL L^− 1^ CaPK, T8: 16 mL L^− 1^ CaPK, T9: 8 mL L^− 1^ CaPK+ salt and T10: 16 mL L^− 1^ CaPK+ salt. Results are shown as a mean of 3 replicates, the bars on the column show ± SE. The values with same letter are non-significant and the value with different letters is significant

Salt stress elevated malondialdehyde (MDA) and H_2_O_2_ levels in bean plants by 112.7% and 140.6%, respectively (Fig. 2), indicating increased lipid peroxidation and oxidative stress. However, foliar application of CaPK fertilizers significantly reduced these levels in stressed and unstressed plants, with bulk treatments being more effective.

### Effect of bulk CaPK and CaPK NPs on mineral uptake of the faba bean plant

The ionic uptake, including Na, K, P, N, and Ca contents, was determined in bean plants grown in either saline or normal soils and sprayed with various levels of bulk and nano fertilizers (Tables 5 and 6). The present data revealed that saline stress stimulated a decline in N, P, K, and Ca contents as well as the K/Na ratio. The percentage of reduction reached about 61.0%, 75.3%, 61.1%, and 66.5%, respectively, compared to unstressed plants. On the contrary, Na content was significantly accumulated in the stressed bean plants (Table 6). Treated stressed plants showed significant increases in N, P, K, and Ca content, as well as an improved K⁺/Na⁺ ratio, alongside a marked reduction in Na⁺ accumulation relative to untreated controls. These effects were most prominent at the 8 mL L^−1^application rate (Tables 5 and 6).

**Table 5:** Impact of CaPK nanoparticles and/or bulk material on mineral profile as K, Na and K/Na ratio of faba bean plants grown in sandy soil. Results are shown as a mean of 3 replicates ± SE. The values with same letter in the same column are non-significant and the value with different letters is significant

Parameters	K mg100 g^− 1^ DW	Na mg 100 g^− 1^ DW	K/Na
Treatments			
Control (normal soil)	36.17 ± 0.67^a^	19.52 ± 0.39^i^	1.88 ± 0.07^a^
Salt Stress (sandy soil)	22.13 ± 0.92^j^	30.4 ± 0.74^a^	0.75 ± 0.05^h^
8 mL L^− 1^ NPs	30.9 ± 2.98^d^	23.69 ± 2.24^f^	1.43 ± 0.26^d^
16 mL L^− 1^ NPs	29.31 ± 2.79^e^	25.45 ± 2.21^e^	1.25 ± 0.22^e^
8 mL L^− 1^ NPs + salt	24.25 ± 0.34^h^	28.69 ± 0.03^b^	0.85 ± 0.01^g^
16 mL L^− 1^ NPs + salt	23.07 ± 0.26^i^	30.39 ± 0.14^a^	0.76 ± 0.01^h^
8 mL L^− 1^ CaPK	33.72 ± 3.04^b^	21.28 ± 2.07^h^	1.74 ± 0.31^b^
16 mL L^− 1^ CaPK	32.04 ± 3.0^c^	23.39 ± 2.12^g^	1.49 ± 0.26^c^
8 mL L^− 1^ CaPK + salt	26.93 ± 0.33^f^	25.9 ± 0.26^d^	1.04 ± 0.02^f^
16 mL L^− 1^ CaPK +salt	25.29 ± 0.2^g^	28.13 ± 0.29^c^	0.9 ± 0.01^g^

**Table 6:** Impact of CaPK nanoparticles and/or bulk material on mineral profile as N, P and Ca ratio of faba bean plants grown in sandy soil. Results are shown as a mean of 3 replicates ±SE. The values with same letter in the same column are non-significant and the value with different letters is significant

Parameters	Nmg100 g^-1^ DW	Pmg 100 g^-1^ DW	Camg 100 g^-1^ DW
Treatments			
Control (normal soil)	32.62±0.93^a^	2.27±0.03^a^	37.02±1.04^a^
Salt Stress (sandy soil)	19.93±0.43^i^	1.71±0.02^d^	24.62±0.99^j^
8 mL L^-1^ NPs	28.73±3.04^c^	2.05±0.12^b^	33.23±1.43^d^
16 mL L^-1^ NPs	26.07±2.83^e^	1.97±0.12^bc^	31±1.75^e^
8 mL L^-1^ NPs + salt	21.94±0.24^g^	1.78±0.01^d^	30.04±0.24^f^
16 mL L^-1^ NPs + salt	19.75±0.23^j^	1.69±0.01^d^	27.11±0.33^i^
8 mL L^-1^ CaPK	29.76±3.42^b^	2.17±0.13^a^	33.86±2.83^c^
16 mL L^-1^ CaPK	27.95±3.32^d^	2.06±0.13^b^	36.68±4.28^b^
8 mL L^-1^ CaPK + salt	22.13±0.29^f^	1.89±0.01^c^	27.57±0.51^g^
16 mL L^-1^ CaPK +salt	20.52±0.06^h^	1.76±0.01^d^	27.13±0.29^h^

### Effect of bulk CaPK and nano CaPKNPs on the oxidative defense system of salt-stressed faba bean plant

**Fig. 3:**
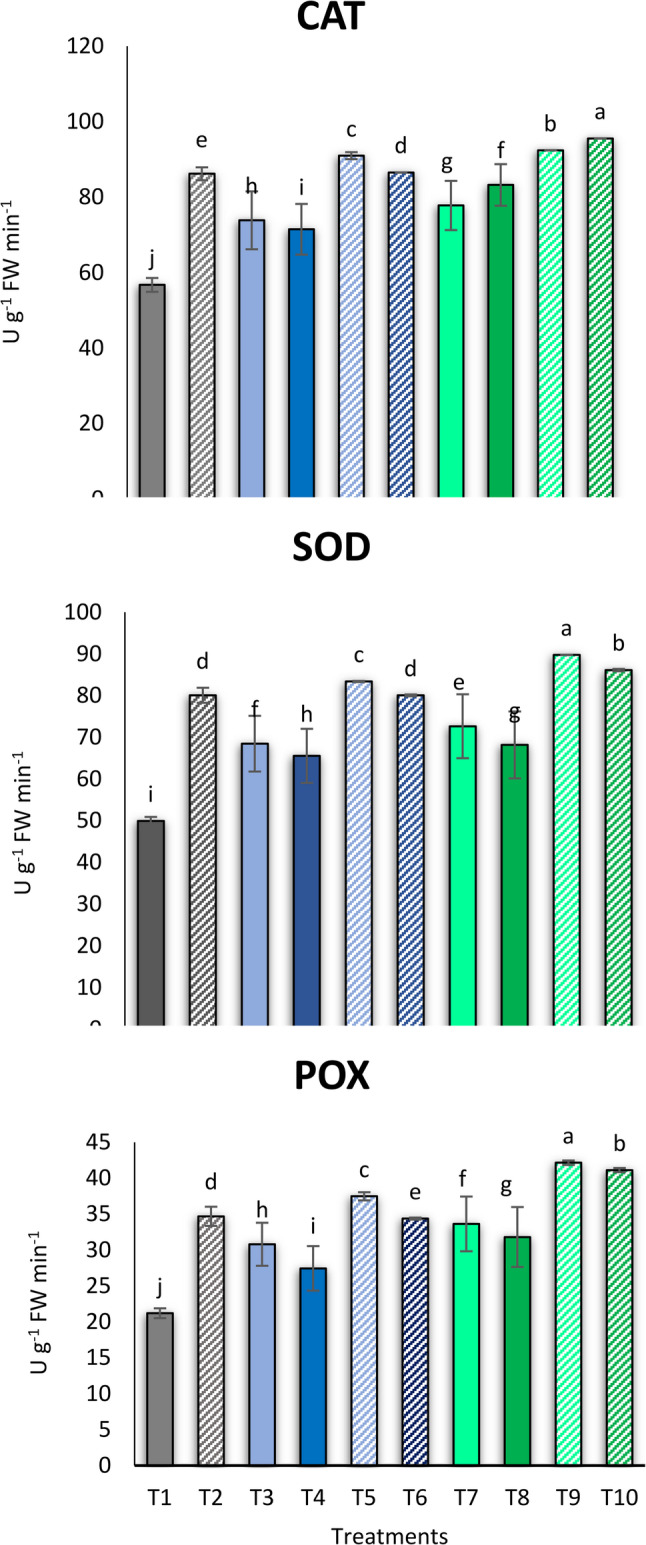
Impact of CaPK nanoparticles and/or bulk material on catalase (CAT), peroxidase (POX) and superoxide dismutase (SOD) activities of faba bean plants grown in sandy soil. T1: control (normal soil), T2: salt stress (sandy soil), T3: 8 mL L^− 1^ NPs, T4: 16 mL L^− 1^ NPs, T5: 8 mL L^− 1^ NPs+ salt, T6: 16 mL L^− 1^ NPs+ salt, T7: 8 mL L^− 1^ CaPK, T8: 16 mL L^− 1^ CaPK, T9: 8 mL L^− 1^ CaPK+ salt and T10: 16 mL L^− 1^ CaPK+ salt. Results are shown as a mean of 3 replicates, the bars on the column show ± SE. The values with same letter are non-significant and the value with different letters is significant

In this study, salt-stressed bean plants exhibited a significant augmentation in antioxidant enzyme activities (Fig. 3), including SOD, CAT, and POX, which increased by about 160.3%, 151.9%, and 163.6%, respectively, compared to unstressed controls. Foliar application of bulk CaPK and CaPK-NPs induced further activations, particularly at 8 mL L^−1^CaPK bulk materials.

### Effect of bulk CaPK and CaPKNPs on total soluble sugar, total soluble protein, proline and total phenols of salt-stressed bean plant

**Fig. 4:**
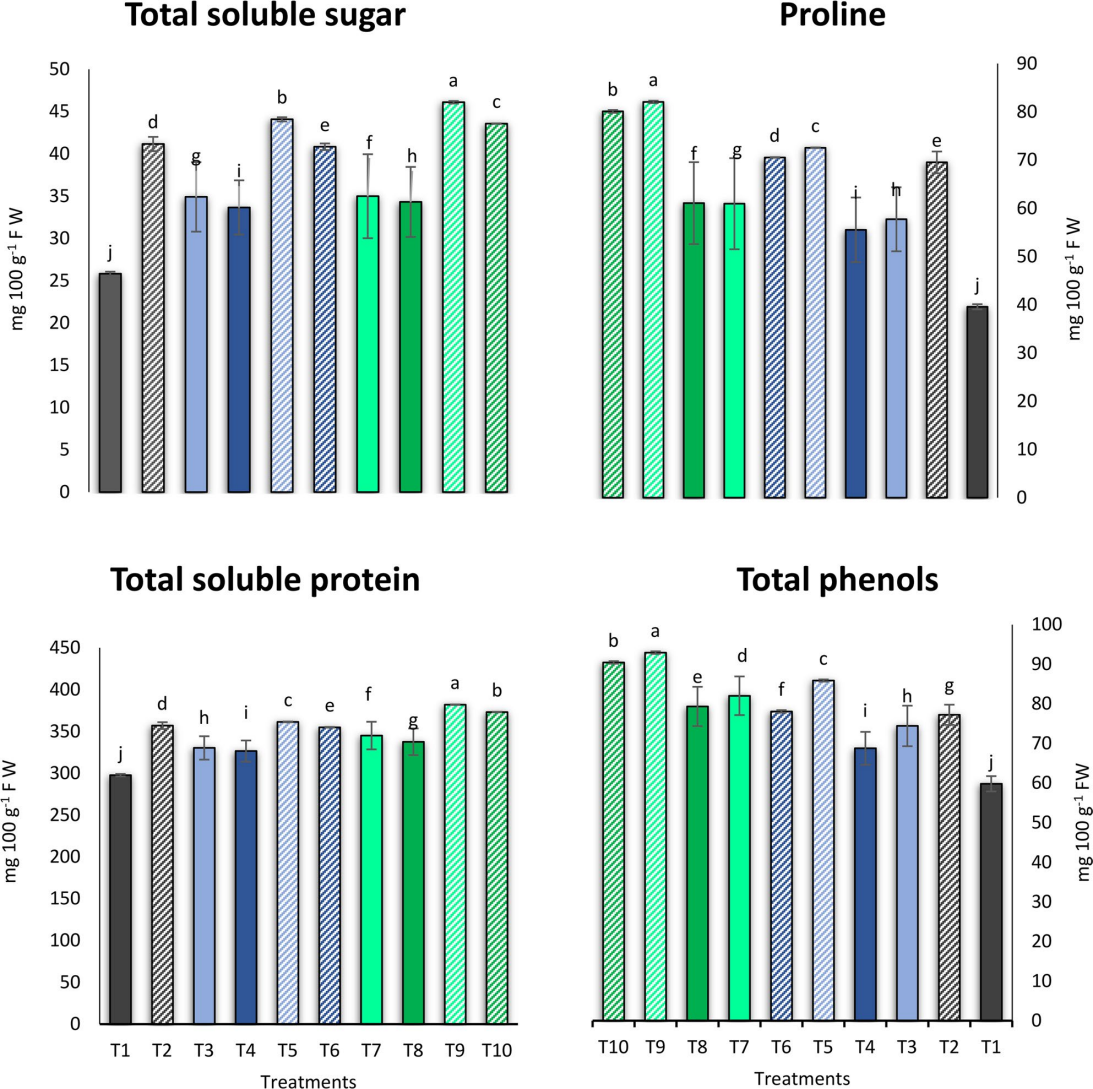
Impact of CaPK nanoparticles and/or bulk material on total soluble sugar, total soluble protein, proline and total phenols contents of faba bean plants grown in sandy soil. T1: control (normal soil), T2: salt stress (sandy soil), T3: 8 mL L^− 1^ NPs, T4: 16 mL L^− 1^ NPs, T5: 8 mL L^− 1^ NPs+ salt, T6: 16 mL L^− 1^ NPs+ salt, T7: 8 mL L^− 1^ CaPK, T8: 16 mL L^− 1^ CaPK, T9: 8 mL L^− 1^ CaPK+ salt and T10: 16 mL L^− 1^ CaPK+ salt. Results are shown as a mean of 3 replicates, the bars on the column show ± SE. The values with same letter are non-significant and the value with different letters is significant

The present study showed that the total soluble sugar level was significantly augmented in faba bean (cv. Misr 3) plants grown in salty soil relative to unstressed plants (Fig. 4). The foliar application of bean plants with different levels of bulk CaPK fertilizers mostly induced a pronounced accumulation of total soluble sugar concentration under unstressed and stressed conditions (Fig. 4). The greatest level of total soluble sugar was assayed in stressed faba plants treated with 8 mL L^−1^of bulk CaPK and NPs. The total soluble sugar content increased to 111.9% and 107.1% above the control levels in stressed plants treated with bulk CaPK and NPs, respectively.

The current investigation showed a significant boost in total soluble protein contents in the stressed bean plants (Fig. 4). The percentage of increase was about 119.9% above the control value. Furthermore, the total soluble protein in stressed bean plants treated with 8 mL L^−1^and 16 mL L^− 1^ of bulk CaPK fertilizers markedly increased by 106.9% and 104.5% respectively, compared to the control values. The greatest increase in the total soluble protein was assayed in plants treated with 8 mL L^−1^of both types of fertilizers.

Furthermore, salt stress significantly stimulated the augmentation in proline content in faba bean plants in comparison to unstressed plants (Fig. 4). The percentage increase was 175.6% above the control value. Further accumulation was displayed by applying both types of fertilizers. Such an effect was more pronounced in bulk CaPK treatment rather than in CaPK nano fertilizers.

Likewise, proline, the application of bulk CaPK fertilizer at different levels also improved significantly the accumulation of total phenols in salt-stressful bean plants by 120.3%, and 117.1% at 8 and 16 mL L^− 1^, respectively, as compared to their corresponding controls (Fig. 4). The greatest phenolics level was attained in stressed- bean plants sprayed with 8 mL L^− 1^ of bulk CaPK fertilizer. In addition, CaPK NPs fertilizers application, results in proline and total phenols accumulation particularly at 8 mL L^− 1^ level (Fig. 4).

### Effect of bulk CaPK and CaPKNPs on gene expression of the faba bean plant

**Fig. 5:**
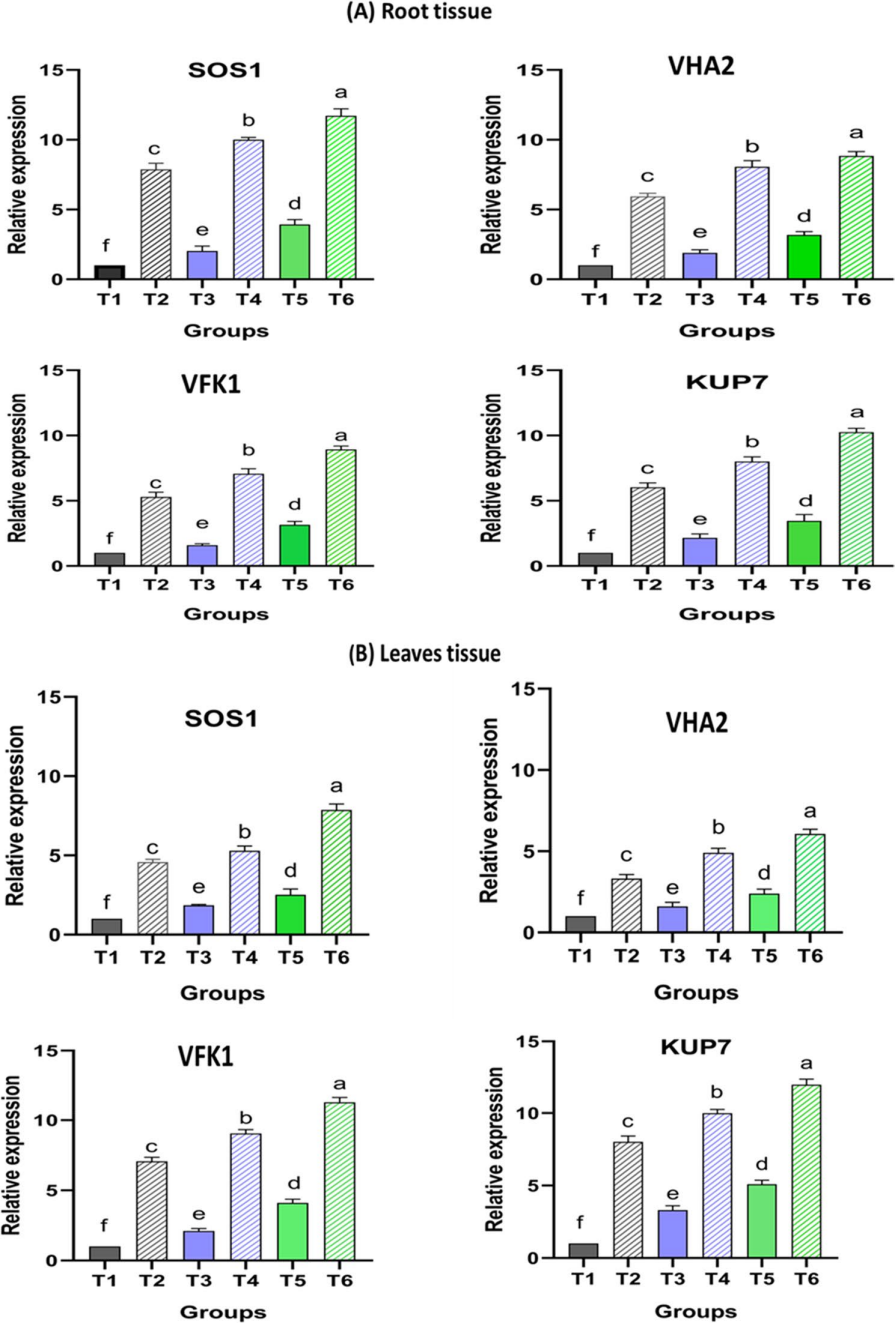
Impact of bulk CaPK and CaPK nanoparticles NPs (8 mL L^− 1^) on the relative expression of membrane ion channel and transporter in *Vicia faba*: *SOS1*,* VHA2*,* VFK1*, and *KUP7*, of faba beans root (A) and leaves (B) under salt stress. T1: control (normal soil), T2: salt stress (sandy soil), T3: 8 mL L^− 1^ NPs, T4:8 mL L^− 1^ NPs+ salt, T5:8 mL L^− 1^ CaPK, T6:8 mL L^− 1^ CaPK+ salt. Data are expressed as mean ± SD. Different letters on data bars indicate significant differences among the treatments at *P* ≤ 0.05

The RT-PCR analysis revealed that salt stress stimulated the overexpression levels of *SOS1(7.89-fold)*,* VFK1(5.30-fold)*,* KUP7(6.03-fold)*, and *VHA2(5.94-fold)* in stressed roots relative to the corresponding unstressed controls (Fig. 5). Additionally, salt stress stimulated the overexpression levels of *SOS1(4.58-fold)*,* VFK1(7.10-fold)*,* KUP7(8.02-fold)*,* and VHA2(3.3-fold)* in stressed leaves relative to the corresponding unstressed controls (Fig. 5). Furthermore, the present results indicated that foliar application of either bulk CaPK or NPs at a concentration of 8 mL L^−1^induced a significant increase in the expression levels of *SOS1*,* VFK1*,* KUP7*, and *VHA2* in both stressed roots and leaves relative to the corresponding unstressed treated controls (Fig. 5). Notably, the greatest expression in these genes was displayed in CaPK bulk-stressed roots and leaves.

## Discussion

Salinity represents a critical abiotic stress that expressively impairs crop yield by limiting water and nutrient absorption as well as Na toxicity [48, 49]. The current data revealed a remarkable reduction in all measured growth parameters and yield components of faba bean cultivar (Misr 3) grown in saline soil (Tables 2 and 3). Such negative impacts of salinity may be attributed to a decline in photosynthetic rate associated with the reduction in chlorophyll a &b contents (Table 4). Salinity stress disrupts plant physiology by inducing ionic and osmotic imbalances that lead to excessive production of reactive oxygen species (ROS), which damage cellular components and impair plant viability [50–52]. From the outcome of the obtained results, the significant decline in the photosynthetic pigments in faba leaves was associated with the accumulation of ROS such as H_2_O_2_ (Fig. 2) which stimulated the chlorophyll oxidative degradation. Notable, the observed damage was evidenced by increased malondialdehyde (MDA) and H_2_O_2_ levels in stressed bean plants (Fig. 2). Furthermore, the reduction in chlorophyll content is also associated with elevated Na⁺ levels, osmotic imbalance, and decreased K⁺ uptake (Table 5), which damage the photosynthetic apparatus and consequently stimulated the disruptions in biochemical and physiological processes which may inhibit cell division, expansion, and thereby the overall plant growth [53, 54].

On the other hand, foliar application of bulk CaPK material at both concentrations 8 mL L^−1^and 16 mL L^− 1^ and CaPK nano fertilizers at 16 mL L^− 1^ induced a marked and substantial increase in all growth and yield parameters in both unstressed and stressed faba bean plants (Tables 2 and 3). The optimum yield performance in both unstressed and salt-stressed faba bean plants was obtained with 16 mL L⁻¹ bulk CaPK (Table 3).These positive effects can be attributed to the essential roles of Ca, K, and P in membrane stabilization, osmotic regulation, enzymatic activation, and ATP synthesis [55, 56]. Phosphorus availability is particularly critical under saline conditions, where metabolic energy demand increases to sustain stress-response mechanisms. Calcium enhances membrane selectivity and structural integrity, while potassium regulates stomatal conductance and maintains cytosolic ion balance [57, 58].

However, the greatest superior performance of bulk CaPK suggests that rapid solubility and immediate nutrient availability may be significant for mitigating stress during sensitive growth stages.

The current data showed that nano-formulated CaPK exhibited a concentration-dependent response. Nanofertilizers possess high surface area and improved penetration efficiency, which may enhance nutrient bioavailability and stress mitigation [59, 60]. Their role in stabilizing membranes, supporting osmotic adjustment, and maintaining protein synthesis and thereby contributed to improved physiological performance. The enhanced chlorophyll content and photosynthetic activity following nanoparticle application was measured in unstressed and stressed faba bean plants as similarly reported in tomato, wheat, and spinach [61–63]. However, excessive nanoparticle concentrations reduced pigment content, consistent with reports of nanoparticle-induced phytotoxicity at higher doses [64]. Such responses reflect that the optimal nanofertilizer concentrations stimulate growth while excessive levels impair cellular function [65]. The consistent superiority of 8 mL L⁻¹ over 16 mL L⁻¹ suggests that moderate foliar concentrations may optimize nutrient uptake efficiency while avoiding excessive ion accumulation on leaf surfaces, which could otherwise disturb cellular homeostasis or induce mild oxidative stress. Thus, the term ‘optimal’ in this study refers to the most effective concentration under the tested conditions rather than a universal recommendation.

In addition, the current results revealed that foliar application of both bulk CaPK fertilizers and CaPK nanofertilizers succeed to alleviate the oxidative stress stimulated by salinity stress as evidenced by reduced MDA and H₂O₂ levels and enhanced antioxidant enzyme activities (SOD, CAT, and POX) in faba been plants (Figs. 2 and 3). This increase suggests enhanced ROS detoxification capacity and maintaining redox homeostasis under salinity [66, 67]. Consequently the photosynthetic pigments increased in both concentrations of bulk CaPK and low concentration only of nanofertilizers (Table 4). This result parallels the results of Wang et al. [64], who reported that high levels of Zn-NPs (> 50%) in *Arabidopsis* reduced chlorophyll content and photosynthetic performance by over 50%, highlighting the potential phytotoxic effects of excessive nanoparticle application. Engineered nanoparticles have been shown to stimulate the antioxidant enzymes system and protect chloroplast integrity under stress conditions [68, 69]. Similar reductions in lipid peroxidation and improvements in membrane stability have been reported with TiO₂ nanoparticles in maize [70]. These findings suggest that CaPK formulations enhance stress tolerance by reinforcing cellular antioxidant capacity.

Salinity also induced the accumulation of soluble sugars, proteins, and proline in faba plants (Fig. 4), reflecting activation of osmoprotective mechanisms. Soluble sugars function as compatible osmolytes that maintain cellular turgor and stabilize macromolecules under dehydration stress [71, 72]. In addition, the further accumulation of soluble sugar of stressed faba plants following fertilizer (bulk and NPs) application (Fig. 4) indicates improved osmotic adjustment. These results were consistent with previous findings in faba bean treated with nanoparticle-based fertilizers [73]. Proline accumulation further contributes to osmotic balance, membrane stabilization, and ROS scavenging [74, 75]. Phenolic compounds also increased under salinity and fertilizer treated faba plants (Fig. 4), supporting their role as non-enzymatic antioxidants capable of neutralizing ROS and limiting their oxidative damage [76]. Nevertheless, interactions between nanoparticles and secondary metabolism require careful evaluation, as excessive stimulation may indicate oxidative signaling rather than cordially beneficial responses [77].

Furthermore, ion imbalance is a symbol of salinity stress. In this study, Na⁺ accumulation increased while N, P, K, and Ca levels declined, leading to a reduced K⁺/Na⁺ ratio (Tables 5 and 6). Foliar application of CaPK and CaPK-NPs restored nutrient balance, improved K⁺ level, and reduced Na⁺ accumulation in faba plants (Table 5). The improved Na⁺/K⁺ ratio reflects better ionic homeostasis, which is directly associated with improved growth stability under salt stress [78]. Nanofertilizers may facilitate nutrient uptake through improved bioavailability and transporter activation [79]. The sustained nutrient delivery and enhanced phosphorus uptake are particularly advantageous in saline soils, where P availability is often limited. Moreover, Ca and P application promotes Na⁺ sequestration into vacuoles and supports cytosolic K⁺ stability, reinforcing cellular homeostasis [80].

At the molecular level, it should be clarified that primer amplification efficiencies were not experimentally determined, so the conclusions drawn from the expression data for *SOS1*,* VHA2*,* KUP7*, and *VFK1* are supported primarily by physiological and biochemical results. Salinity increased the expression level of ion transporter genes, including *SOS1*, *VHA2*, *KUP7*, and *VFK1* (Fig. 5), consistent with their recognized roles in Na⁺ extrusion, vacuolar sequestration, and K⁺ transport [81]. *SOS1-*mediated Na⁺/H⁺ exchange is essential to Na⁺ exclusion and salt tolerance [82], while *VHA2* generates proton gradients necessary for vacuolar Na⁺compartmentation and osmotic adjustment [83]. *KUP7* and *VFK1* facilitate K⁺ uptake and redistribution, maintaining optimal cytosolic K⁺/Na⁺ ratios essential for enzymatic function and stress resilience [84, 85]. The enhanced expression of these genes in plants treated with low concentrations of CaPK was associated with improved ion homeostasis and reduced oxidative damage, thereby inducing the physiological and transcriptional regulation. But higher concentrations of CaPK formulations, particularly in nano form, yielded negative physiological results, so gene expression analyses were limited to low concentrations. High concentrations may induce aggregation or ionic imbalance, limiting physiological efficiency. Maintaining optimal ion ratios rather than increasing absolute nutrient concentrations appears fundamental for effective salinity mitigation [86].

Notably, bulk fertilizers may produce superior results in faba bean (*Vicia faba*) because they release nutrients in easily soluble ionic form that are immediately available for plant uptake. These mineral salts (bulk) may enhance nutrient diffusion toward roots or facilitate faster foliar absorption, which better matches crop demand. As plant development progresses, nutrient uptake and partitioning patterns change substantially. Accordingly, the application of bulk fertilizer at 8 mL L⁻¹ produced the most favorable physiological, biochemical, and gene expression responses during the early growth stage, whereas the highest yield parameters were obtained at 16 mL L⁻¹. This difference may be attributed to the increased demand for phosphorus and potassium during the later stages of plant development, as these nutrients are required in greater amounts to support reproductive growth and yield formation [87].

In contrast, nanoforms may show slower release, particle aggregation, or interactions with leaf surfaces that limit nutrient availability. Therefore, the direct availability of nutrients in bulk salt form can sometimes result in more pronounced growth and yield responses compared with nanoformulations.

Consequently, the integration of physiological, biochemical, and gene expression evidence demonstrates that both bulk and nano CaPK enhance salt tolerance by improving nutrient acquisition, strengthening antioxidant defense systems, supporting osmotic adjustment, and regulating ion transport pathways. Future research should also evaluate long-term environmental impacts and nanoparticle persistence to ensure sustainable application in saline agroecosystems.

## Conclusion

This study demonstrated that calcium–potassium phosphate (CaPK) fertilizers, in both bulk and nanoparticle forms, enhance salinity tolerance in faba bean through coordinated physiological, biochemical, and molecular responses. Bulk CaPK treatments consistently improved nutrient uptake, photosynthetic performance, osmolyte accumulation, and antioxidant activity under saline conditions. Both fertilizer forms were associated with the upregulation of selected ion transport-related genes (*SOS1*,* VHA2*, *KUP7*, and *VFK1*), contributing to Na⁺ exclusion, K⁺ retention, and vacuolar ion sequestration, thereby maintaining ionic homeostasis. Although nanoparticle formulations offer the advantage of controlled nutrient release, bulk CaPK generally produced more rapid and consistent improvements in plant growth and yield. This finding further indicates that fertilizer efficiency depends on matching nutrient supply with crop requirements throughout different developmental stages. Foliar application of bulk CaPK at 8 mL L⁻¹ was most effective during the early vegetative stage, resulting in superior physiological, biochemical, and molecular responses, whereas 16 mL L⁻¹ was optimal during the reproductive stage, leading to the highest yield performance. Overall, these results underscore the practical value of bulk CaPK as an effective strategy for mitigating salinity stress and improving the productivity of faba bean cultivated under saline conditions.

## Supplementary Information

Supplementary Material 1

## Data Availability

All data generated or analyzed during this research are fully included in the published article.

## Abbreviations

CaPK: Calcium–phosphate–potassium bulk
CaPK-NP: Calcium–phosphate–potassium nanoparticle
CAT: Catalase
Chl a: Chlorophyll a
Chl b: Chlorophyll b
VHA2: Ion transporter genes –ATPase
KUP7: Potassium uptake permease 7 gene
MDA: Malondialdehyde
POX: Peroxidase
ROS: Reactive oxygen species
SOD: Superoxide dismutase
SOS1: Salt overly sensitive1gene
VFK1: Potassium channel1gene

## References

[1] Alsulami N. Major abiotic factors affecting plants. JKAU Sci. 2024;34(2).

[2] Wang X, Li Z, Hu Z. Alleviation of plant abiotic stress: mechanistic insights into phosphate-solubilizing microorganisms in agriculture. Plants (Basel). 2025;14(10):1558, 1558. 40431124 10.3390/plants14101558 PMC12115179

[3] FAO. Global assessment of salt-affected soils. Rome: Food and Agriculture Organization of the United Nations; 2021.

[4] Shrivastava P, Kumar R. Soil salinity: a serious environmental issue and plant growth promoting bacteria as one of the tools for its alleviation. Saudi J Biol Sci. 2015;22:123–31. 25737642 10.1016/j.sjbs.2014.12.001 PMC4336437

[5] Punia H, Tokas J, Malik A, Singh S, Phogat DS, Bhuker A, et al. Discerning morpho-physiological and quality traits contributing to salinity tolerance acquisition in sorghum [Sorghum bicolor (L.) Moench]. S Afr J Bot. 2021;140:409–18.

[6] El-Awadi ME, Sadak MS, Khater MA, Dawood MG. Melatonin stimulates salt tolerance of soybean plants by modulating photosynthetic performance, osmoregulation, and the enzymatic antioxidant defence system. Acta Biol Slov. 2025; 68(3).

[7] Junedi MA, Mukhopadhyay R, Manjari KS. Alleviating salinity stress in crop plants using new engineered nanoparticles (ENPs). Plant Stress. 2023;9:100184.

[8] El-Lethy SR, Sadak MS, Hanafy RS. Assessing the usefulness of *Moringa oleifera* leaf extract and zeatin in enhancing growth, phytohormones, antioxidant enzymes and osmoprotectants of wheat plant under salinity stress. Egypt J Bot. 2024;64(3):183–96.

[9] Rhaman MS, Rauf F, Tania SS, et al. Proline and glycine betaine: a dynamic duo for enhancing salt stress resilience in maize by regulating growth, stomatal size, and oxidative stress responses. Plant Stress. 2024;14:100563.

[10] Sadak MS, Dawood MG, El-Awadi MES. Changes in growth, photosynthetic pigments and antioxidant system of *Hordeum vulgare* plant grown under salinity stress via signal molecules application. Vegetos. 2024. 10.1007/s42535-024-00879-3.

[11] Shi H, Bressan R, Hasegawa PM, Zhu JK. Sodium. In: Broadley M, White P, editors. Plant nutritional genomics. London: Blackwell; 2005. p. 127–49.

[12] Wang Y, Wu WH. Potassium transport and signaling in higher plants. Annu Rev Plant Biol. 2013;64:451–76. 23330792 10.1146/annurev-arplant-050312-120153

[13] Roy S, Mishra M, Dhankher OP. Molecular chaperones: key players of abiotic stress response in plants. In: Genetic enhancement of crops for tolerance to abiotic stress: mechanisms and approaches. Cham: Springer; 2019. p. 125–65.

[14] Mohamed EA, Osama E, Manal E, et al. Impact of gamma irradiation pretreatment on biochemical and molecular responses of potato growing under salt stress. Chem Biol Technol Agric. 2021;8:35.

[15] Sattari SZ, Bouwman AF, Giller KE, Van Ittersum MK. Residual soil phosphorus as the missing piece in the global phosphorus crisis puzzle. Proc Natl Acad Sci USA. 2012;109:6348–53. 22431593 10.1073/pnas.1113675109 PMC3341047

[16] Dimkpa CO, Bindraban PS. Nanofertilizers: new products for the industry? J Agric Food Chem. 2017;65:4640–6. 10.1021/acs.jafc.7b02150 28535672

[17] Khan MN, Mobin M, Abbas ZK, AlMutairi KA. Zinc oxide nanoparticles improve growth, antioxidant response, and yield of crops under stress conditions. Environ Sci Pollut Res. 2019;26:21521–35.

[18] Latef AAHA, Srivastava AK, El-Sheikh MA, et al. Silicon nanoparticles improve salt tolerance in plants. Plant Physiol Biochem. 2017;110:122–32.

[19] El-Sheikh MA, et al. Role of nanoparticles in alleviating salinity stress in plants. Plant Physiol Biochem. 2021;159:70–80.

[20] Rastogi A, Zivcak M, Sytar O, et al. Impact of metal and metal oxide nanoparticles on plant physiology. Plant Physiol Biochem. 2019;132:148–57.

[21] Cele T. Preparation of nanoparticles. Eng Nanomater Heal Saf. 2020. 10.5772/intechopen.90771.

[22] Zeidan MS. Effect of sowing dates and urea foliar application on growth and seed yield of determinate faba bean (*Vicia faba* L.) under Egyptian conditions. Egypt J Agron. 2002;24:93–102.

[23] El-Husseiny AAH, Nada WM, Mahrous H. Improving chemical and microbial properties of calcareous soil and its productivity of faba bean (*Vicia faba* L.) plants by using compost tea enriched with humic acid and Azolla. Egypt J Soil Sci. 2020;61(1):27–44.

[24] Katerji N, Van Hoorn JW, Hamdy A, Mastrorilli M. Salinity effect on crop development and yield, analysis of salt tolerance according to several classification methods. Agric Water Manag. 2003;62(1):37–66.

[25] Ashraf MF, Foolad MR. Roles of glycine betaine and proline in improving plant abiotic stress resistance. Environ Exp Bot. 2007;59(2):206–16.

[26] El-Sayed MA, et al. Performance and salinity tolerance assessment of faba bean (*Vicia faba* L.) cultivars under Egyptian conditions. Egypt J Agron. 2019;41(2):195–206.

[27] Bianco A, Cacciotti I, Lombardi M, et al. Thermal stability and sintering behaviour of hydroxyapatite nanopowders. J Therm Anal Calorim. 2007;88:237–43.

[28] Chapman HD, Pratt PF. Method of analysis for soil, plants and waters. Berkeley: Univ California, Agricultural Science; 1978. p. 4034.

[29] Metzner H, Rau H, Senger H. Untersuchungen zur synchronisierbarkeit einzelner pigmentmangel-mutanten von Chlorella. Planta. 1965;65:186–94.

[30] Homme PM, Gonzalez B, Billard J. Carbohydrate content, fructan and sucrose enzyme activities in roots, stubble and leaves of rye grass (*Lolium perenne* L.) as affected by source/sink modification after cutting. J Plant Physiol. 1992;140:282–91.

[31] Blakeney AB, Mutton LL. A simple colorimetric method for the determination of sugar in fruit and vegetables. J Sci Food Agric. 1980;31:889–97.

[32] Mæhre HK, Jensen IJ, Eilertsen KE. Enzymatic pre-treatment increases the protein bioaccessibility and extractability in dulse (*Palmaria palmata*). Mar Drugs. 2016;14(11):196, 196. 10.3390/md14110196. 27792166 10.3390/md14110196 PMC5128739

[33] Bates LS, Waldren RP, Teare ID. Rapid determination of free proline for water-stress studies. Plant Soil. 1973;39:205–7.

[34] Minotti G, Aust SD. The requirement for iron (III) in the initiation of lipid peroxidation by iron (II) and hydrogen peroxide. J Biol Chem. 1987;262:1098–104. 3027077

[35] Malik CP, Singh MB. Plant enzymology and histoenzymology. New Delhi: Kalyani; 1980. p. 286.

[36] Yu CW, Murphy TM, Lin CH. Hydrogen peroxide-induces chilling tolerance in mung bean mediated through ABA-independent glutathione accumulation. Funct Plant Biol. 2003;30:955–63. 32689080 10.1071/FP03091

[37] Mukherjee SP, Choudhuri MA. Implication of water stress-induced changes in the level of endogenous ascorbic acid and hydrogen peroxide in vigna seedling. Physiol Plant. 1983;58:166–70.

[38] Marklund S, Marklund G. Involvement of the superoxide anion radical in the autoxidation of pyrogallol and a convenient assay for superoxide dismutase. Front Plant Sci. 1974;47(3):469–74. 10.1111/j.1432-1033.1974.tb03714.x 4215654

[39] Chen Y, Cao XD, Lu Y, Wang XR. Effects of rare earth metal ions and their EDTA complexes on antioxidant enzymes of fish liver. Bull Environ Contam Toxicol. 2000;65:357–65. 10903360 10.1007/s0012800136

[40] Kar M, Mishra D. Catalase, peroxidase and polyphenol oxidase activities during rice leaf senescence. Plant Physiol. 1976;57:315–9. 16659474 10.1104/pp.57.2.315 PMC542015

[41] Chapman HD, Pratt PF. Method of analysis for soil, plants and waters. Berkeley: Univ California, Agricultural Science; 1961. p. 309.

[42] AOAC, Rosenthal A. Official methods of analysis. Association of official analytical chemist international. 17th ed. Pyle: Gaithersburg MD; USA; 2002.

[43] Page AL. Methods of soil analysis, part 2: chemical and microbiological properties. 2nd ed. Madison: SSSA Inc; 1982.

[44] Jackson ML. Soil chemical analysis. New Delhi: Prentice Hall of India Pvt. Ltd; 1973.

[45] Sagervanshi A, Naeem A, Geilfus CM, Geilfus C, et al. One-time abscisic acid priming induces long-term salinity resistance in *Vicia faba*: changes in key transcripts, metabolites, and ionic relations. Physiol Plant. 2020;172:146–61. 33314239 10.1111/ppl.13315

[46] Livak KJ, Schmittgen TD. Analysis of relative gene expression data using real time quantitative PCR and the 2a Act method. Methods. 2001;25:402–8. 11846609 10.1006/meth.2001.1262

[47] Casella G. Statistical design. 1st ed. Gainesville: Springer; 2008.

[48] Babitha KC, Ramu S, Karaba N, et al. Overexpression of EcbHLH57 transcription factor from *Eleusinecoracana* L. in tobacco confers tolerance to salt, oxidative and drought stress. PLoS ONE. 2015;10:e0137098. 26366726 10.1371/journal.pone.0137098 PMC4569372

[49] Singh D, Singh CK, Taunk J, Singh V, et al. Transcriptome skimming of lentil (*LensculinarisMedikus*) cultivars with contrast reaction to salt stress. Funct Integr Genomics. 2021. 10.1007/s10142-020-00766-5. 10.1007/s10142-020-00766-5 33389259

[50] Munns R, Tester M. Mechanisms of salinity tolerance. Ann Rev Plant Biol. 2008;59:651–81. 18444910 10.1146/annurev.arplant.59.032607.092911

[51] Singh D, Singh CK, Taunk J, Sharma S, Gaikwad K, Singh V, et al. Transcriptome skimming of lentil (*LensculinarisMedikus*) cultivars with contrast reaction to salt stress. Funct Integr Genomics. 2021. 10.1007/s10142-020-00766-5. 10.1007/s10142-020-00766-5 33389259

[52] Zeeshan M, Wang X, Salam A, Wu H, Li S, Zhu S, et al. Selenium nanoparticles boost the drought stress response of soybean by enhancing pigment accumulation, oxidative stress management and ultrastructural integrity. Agron. 2024;14:1372. 10.3390/agronomy14071372.

[53] Acosta-Motos JR, Ortuño MF, Bernal-Vicente A, Diaz-Vivancos P, Sanchez-Blanco MJ. Hernandez JA Plant responses to salt stress: Adaptive mechanisms. Agron. 2017;7:18.

[54] Yang Z, Li JL, Liu LN, Xie Q, Sui N. Photosynthetic regulation under salt stress and salt-tolerance mechanism of sweet sorghum. Front Plant Sci. 2020;10. 10.3389/fpls.2019.01722. 10.3389/fpls.2019.01722 PMC6974683 32010174

[55] Turuko M, Mohammed A. Effect of different phosphorus fertilizer rates on growth, dry matter yield and yield components of common bean (*Phaseolus vulgaris* L.). World J Agric Res. 2014;2(3):88–92.

[56] El-Azizy FA, Habib AAM, Abd El-Baset AM. Effect of nano phosphorus and potassium fertilizers on productivity and mineral content of broad bean in north Sinai. J Soil Sci Agric Eng Mansoura Univ. 2021;12:239–46.

[57] Nasrallah AK, Kheder AA, Kord MA, Fouad AS, El-Mogy MM, Atia MA. Mitigation of salinity stress effects on broad bean productivity using calcium phosphate nanoparticles application. Horticulturae. 2022;8(1):75.

[58] Kaya C, Kirnak H, Higgs D. Enhancement of growth and normal growth parameters by foliar application of potassium and phosphorus in tomato cultivars grown at high (NaCl) salinity. J Plant Nutr. 2001;24(2):357–67.

[59] Soliman AS, El-Feky SA, Darwish E. Alleviation of salt stress on Moringa peregrina using foliar application of nanofertilizers. J Hortic For. 2015;7:36–47.

[60] Burhan SA, Al-Hassan MG. Impact of nano NPK fertilizers to correlation between productivity, quality and flag leaf of some bread wheat varieties. Iraqi J Agric Sci. 2019;50:1–7.

[61] Raliya R, Nair R, Chavalmane S, Wang WN, Biswas P. Mechanistic evaluation of translocation and physiological impact of titanium dioxide and zinc oxide nanoparticles on the tomato (Solanum lycopersicum L.) plant. Metallomics. 2015;7:1584–94. 26463441 10.1039/c5mt00168d

[62] Rizwan M, Ali S, Ali B, Adrees M, Arshad M, Hussain A, et al. Zinc and iron oxide nanoparticles improved the plant growth and reduced the oxidative stress and cadmium concentration in wheat. Chemosphere. 2019;214:269–77. 30265934 10.1016/j.chemosphere.2018.09.120

[63] Zafar S, Perveen S, Khan MK, Shaheen MR, Hussain R, Sarwar N, et al. Effect of zinc nanoparticles seed priming and foliar application on the growth and physio-biochemical indices of spinach (*Spinacia oleracea* L.) under salt stress. PLoS ONE. 2022;17:e0263194. 35192615 10.1371/journal.pone.0263194 PMC8863234

[64] Wang X, Yang X, Chen S. Zinc oxide nanoparticles affect biomass accumulation and photosynthesis in Arabidopsis. Front Plant Sci. 2016;6:1243. 26793220 10.3389/fpls.2015.01243 PMC4709445

[65] Xiong T, Zhang S, Kang Z, Zhang T, Li S. Dose-dependent physiological and transcriptomic responses of lettuce (*Lactuca sativa* L.) to copper oxide nanoparticles—insights into the phytotoxicity mechanisms. Int J Mol Sci. 2021;22(7):3688. 33916236 10.3390/ijms22073688 PMC8036535

[66] Guo Q, Liu L, Barkla BJ. Membrane Lipid Remodeling in Response to Salinity. Int J Mol Sci. 2019;20:4264. 31480391 10.3390/ijms20174264 PMC6747501

[67] Negacz K, Vellinga P, Barrett-Lennard E, Choukr-Allah R, Elzenga T. Future of sustainable agriculture in saline environments (1st ed.) CRC Press (1st Edition) CRC Press. 2021, Pp 541.

[68] Almeer RS, El-Khadragy MF, Abdel Moneim AE. The potential role of nanoparticles in the mitigation of abiotic stress in crop plants. Biotechnol Bioprocess Eng. 2018;23(6):438–53.

[69] Mohammadi M, Aelaei M, Saidi M. Pre-harvest and pulse treatments of spermine, γ-and β-aminobutyric acid increased antioxidant activities and extended the vase life of gerbera cut flowers ‘Stanza’. Ornam Hortic. 2020;26:306–16.

[70] Shah T, Latif S, Saeed F, Ali I, Ullah S, Alsahli AA, et al. Seed priming with titanium dioxide nanoparticles enhances seed vigor, leaf water status, and antioxidant enzyme activities in maize (*Zeamays* L.) under salinity stress. J King Saud Univ Sci. 2021;33:101207.

[71] Rolland F, Baena-Gonzalez E, Sheen J. Sugar sensing and signaling in plants: Conserved and novel mechanisms. Ann Rev Plant Biol. 2006;57:675–709. 16669778 10.1146/annurev.arplant.57.032905.105441

[72] Bhattacharya S, Kundu A. Sugars and sugar polyols in overcoming environmental stresses, in protective chemical agents in the amelioration of plant abiotic stress: biochem mol perspec. In: Protective chemical agents in the amelioration of plant abiotic stress: biochemical and molecular perspectives. 2020. p. 71–101.

[73] Nasrallah AK, Kheder AA, Kord MA, Fouad AS, El-Mogy MM, Atia MA. Mitigation of salinity stress effects on broad bean productivity using calcium phosphate nanoparticles application. Horticulturae. 2022;8(1):75.

[74] Wani AS, Ahmad A, Hayat S, Tahir I. Epibrassinolide and proline alleviate the photosynthetic and yield inhibition under salt stress by acting on antioxidant system in mustard. Plant Physiol Biochem. 2019;135:385–94. 30616113 10.1016/j.plaphy.2019.01.002

[75] Rehman AU, Bashir F, Ayaydin F, Kóta Z, Páli T, Vass I. Proline is a quencher of singlet oxygen and superoxide both in in vitro systems and isolated thylakoids. Physiol Plant. 2021;172(1):7–18. 33161571 10.1111/ppl.13265

[76] Michalak A. Phenolic compounds and their antioxidant activity in plants growing under heavy metal stress. Pol J Environ Stud. 2006;15(4):523–30.

[77] Abdal Dayem A, Hossain MK, Lee SB, Kim K, Saha SK, Yang GM, et al. The role of reactive oxygen species (ROS) in the biological activities of metallic nanoparticles. Int J Mol Sci. 2017;18:120–41. 28075405 10.3390/ijms18010120 PMC5297754

[78] Bargaz A, Nassar R, Rady M, Gaballah M, Thompson S, Brestic M, et al. Improved salinity tolerance by phosphorus fertilizer in two Phaseolus vulgaris recombinant inbred lines contrasting in their P-efficiency. J Agron Crop Sci. 2016;202(6):497–507.

[79] Solanki P, Bhargava A, Chhipa H, Jain N, Panwar J. Nano-fertilizers and their smart delivery system. In: Nanotechnologies in Food and Agriculture. Cham, Switzerland: Springer; 2015. p. 81–101.

[80] de Andrade FHA, Pereira WE, Maia JM, Batista MI, Lima JS, Silva VA. Phosphorus increases K^+^ in the shoot and improves salinity tolerance in sweetsop seedlings. J Plant Growth Regul. 2022;41:1229–40.

[81] Sagervanshi A, Naeem A, Geilfus CM. One-time abscisic acid priming induces long-term salinity resistance in *Vicia faba*: Changes in key transcripts, metabolites, and ionic relations. Physiol Plant. 2021;172:146–61. 33314239 10.1111/ppl.13315

[82] Gao J, Jing S, Cao P, Ren L, Chen S, Chen F, Jiang J. Variation in tissue Na^+^ content and the activity of SOS1 genes among two species and two related genera of Chrysanthemum. BMC Plant Biol. 2016;16:98. 27098270 10.1186/s12870-016-0781-9 PMC4839091

[83] Li Y, Hu J, Qi J, Zhao F, Liu J, Chen L. Improvement of leaf K^+^ retention is a shared mechanism behind CeO_2_ and Mn_3_O_4_ nanoparticles improved rapeseed salt tolerance. Stress Biol. 2022;2(1):1–15. 37676336 10.1007/s44154-022-00065-y PMC10441935

[84] Dreyer I, Uozumi N. Potassium channels in plant cells. FEBS Lett. 2011;278:4293–303. 10.1111/j.1742-4658.2011.08371.x 21955642

[85] Li W, Xu G, Abdel A, Ling Y. Plant HAK/KUP/KT K^+^ transporters: function and regulation. Semin Cell Dev Biol. 2017;74:133–41. 28711523 10.1016/j.semcdb.2017.07.009

[86] Singh J, Singh RP, Kaushik AK, Adetunji CO, Sing KR, editors. Nanobiotechnology for food processing and packaging. Elsevier; 2024.

[87] Jones C, Olson-Rutz K, Dinkins C. Nutrient uptake timing by crops. Montana, USA: Montana State University; 2011.

