# Supplementary material for: Comparative evaluation of bulk and nano-formulated calcium, phosphorus, and potassium fertilizers in improving salinity tolerance of faba bean
Source: BMC Plant Biol. 2026 Jun 15;26:1033. doi: 10.1186/s12870-026-09205-2 (PMC13267729; doi:10.1186/s12870-026-09205-2)
Supplement: Supplementary file 1 — Supplementary Material 1 [file 12870_2026_9205_MOESM1_ESM.docx]

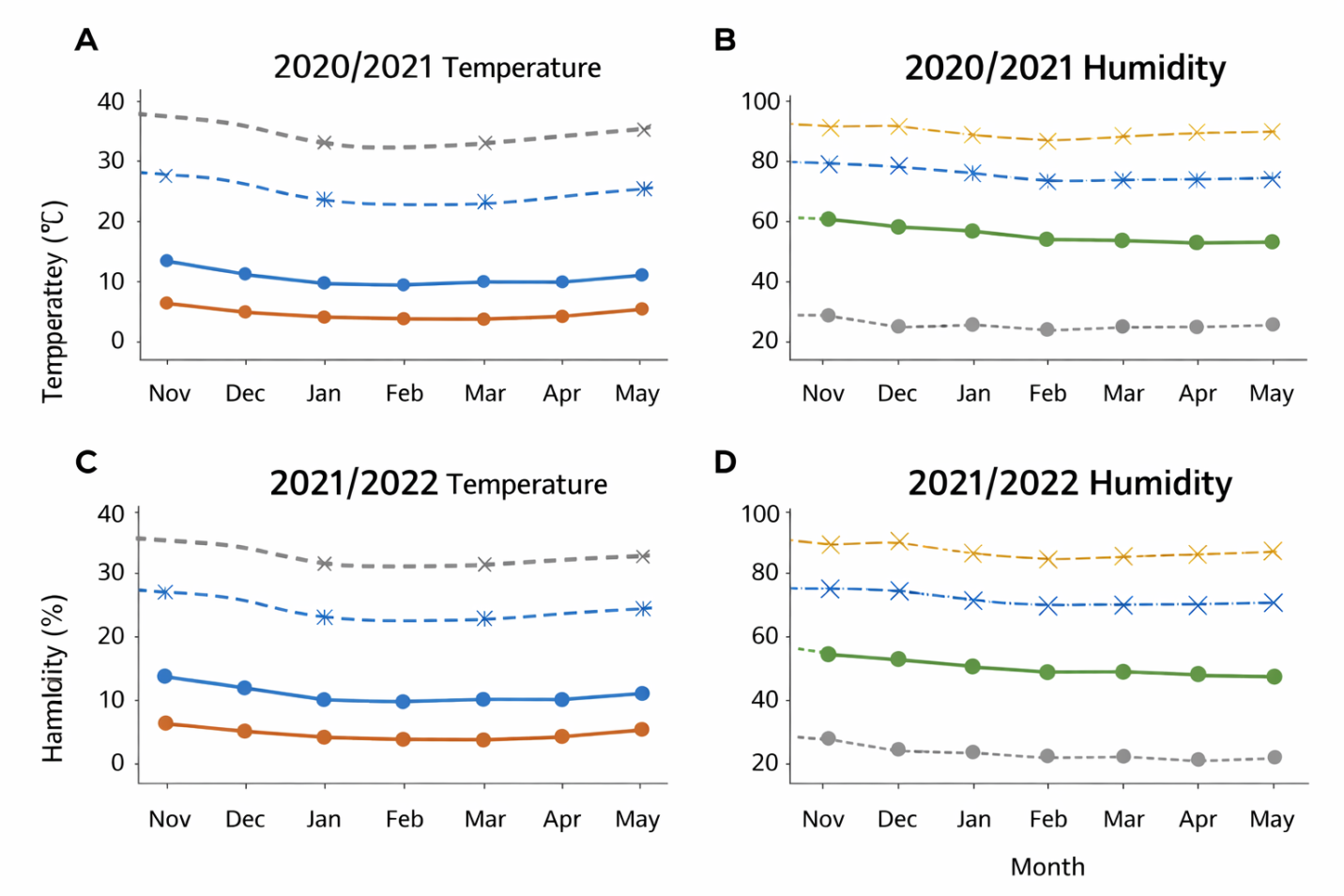


**Figure S1.** Maximum, minimum, and average temperatures, along with relative humidity during the 2020/2021 and 2021/2022 growing seasons at the experimental site (National Research Centre, Nubaria region, Egypt.

**Table S1.** Forward and reverse primer sequences for qPCR.

| Gene name | Primer sequence | GenBank accession number |
| --- | --- | --- |
| *VfSOS1*_fam* | F 5′--GTTCGTGGACCACTTGTAAG-3′  R 5′-GCTCCCATGCGTAAAAGTTG-3′ | [XM020366788.2](https://www.ncbi.nlm.nih.gov/nucleotide/XM_020366788.2?report=genbank&log$=nucltop&blast_rank=8&RID=RBPBJMTZ014)  JQ287499.1  MF405370.1 |
| *VfKUP7*_fam* | F 5′-CTTCCTGGGATTGGAGTTGAC-3′  R 5′-GGAGCTCGTATTGTCCCAAG-3′ | [XM003593148.3](https://www.ncbi.nlm.nih.gov/nucleotide/XM_003593148.3?report=genbank&log$=nucltop&blast_rank=1&RID=5FZF4VZD01R)  XM028349359.1  [XM020366812.2](https://www.ncbi.nlm.nih.gov/nucleotide/XM_020366812.2?report=genbank&log$=nucltop&blast_rank=7&RID=5FZF4VZD01R) |
| *VfVHA2* | F 5′-GTTCCATCTCCCTTACCCGATAG-3′  R 5′-CATGAATCAGGTGCCTAACTCC-3′ | AB022442 |
| *VfVFK1* | F 5′-CCGATGTGCTAGGCTTCTTTC-3′  R 5′-CAGTGGTCATGGTAGTGATGG-3′ | Y10579.1 |
| *VfCYP* | F 5′-TGCCGATGTCACTCCCAGAA-3′  R 5′-CAGCGAACTTGGAACCGTATA-3′ | L32095 |

*Family specific conserved primer.

Whereas, Salt overly sensitive1: SOS; potassium uptake permease 7: KUP7; Ion transporter genes -ATPase: HA2; potassium channel 1: VFK1. Vicia faba cyclophilin: VfCYP

**Table** **S2**. Results of two-way analysis of variance (ANOVA II) showing effects of nanoparticles (NPs), bulk fertilizers, salt, and their interactions, on growth and various physiological parameters and expression of membrane ion channel and transporter in *Vicia faba,* plant.

| Parameters | Source | df | Sum of square | Mean of square | F-value | Significant difference  (P value ˂0.05) |
| --- | --- | --- | --- | --- | --- | --- |
| Plant height at 90 days | Salt Stress | 2 | 101.09 | 50.54 | 680.02 | 0.000^***^ |
|  | Treatment | 3 | 103.17 | 34.39 | 462.66 | 0.000^***^ |
|  | Interaction  (Salt × treatment) | 3 | 7.13 | 2.37 | 31.99 | 0.000^***^ |
| Plant height at harvest | Salt Stress | 2 | 461.41 | 230.70 | 2239.88 | 0.000^***^ |
|  | Treatment | 3 | 174.09 | 58.03 | 563.41 | 0.000^***^ |
|  | Interaction  (Salt × treatment) | 3 | 1.21 | 0.405 | 3.932 | 0.023^*^ |
| Stem weight (g) | Salt Stress | 2 | 549.50 | 274.75 | 2174.80 | 0.000^***^ |
|  | Treatment | 3 | 993.0 | 331.0 | 2620.06 | 0.000^***^ |
|  | Interaction  (Salt × treatment) | 3 | 16.87 | 5.625 | 44.525 | 0.000^***^ |
| Leaf weight(g) | Salt Stress | 2 | 82.66 | 41.33 | 518.83 | 0.000^***^ |
|  | Treatment | 3 | 699.04 | 233.01 | 2924.88 | 0.000^***^ |
|  | Interaction  (Salt × treatment) | 3 | 7.82 | 2.609 | 32.75 | 0.000^***^ |
| Weight of pods/ plant | Salt Stress | 2 | 914.49 | 457.24 | 4916.61 | 0.000^***^ |
|  | Treatment | 3 | 3513.03 | 1171.01 | 12591.51 | 0.000^***^ |
|  | Interaction  (Salt × treatment) | 3 | 33.685 | 11.228 | 120.73 | 0.000^***^ |
| Number of pods/ plant | Salt Stress | 2 | 3.822 | 1.911 | 31.671 | 0.000^***^ |
|  | Treatment | 3 | 103.528 | 34.509 | 571.98 | 0.000^***^ |
|  | Interaction  (Salt × treatment) | 3 | 0.928 | 0.309 | 5.129 | 0.009^**^ |
| Weight of seed/ plant | Salt Stress | 2 | 641.507 | 320.75 | 4202.01 | 0.000^***^ |
|  | Treatment | 3 | 1770.33 | 590.11 | 7730.70 | 0.000^***^ |
|  | Interaction  (Salt × treatment) | 3 | 25.965 | 8.655 | 113.382 | 0.000^***^ |
| 100 – seed weight | Salt Stress | 2 | 33.022 | 16.511 | 262.077 | 0.000^***^ |
|  | Treatment | 3 | 265.68 | 88.560 | 1405.71 | 0.000^***^ |
|  | Interaction  (Salt × treatment) | 3 | 5.752 | 1.917 | 30.432 | 0.000^***^ |
| Seed yield ton/ ha | Salt Stress | 2 | 1.233 | 0.617 | 24.342 | 0.000^***^ |
|  | Treatment | 3 | 0.542 | 0.181 | 7.127 | 0.002^**^ |
|  | Interaction  (Salt × treatment) | 2 | 0.257 | 0.086 | 3.377 | 0.039^*^ |

| Parameters | Source | df | Sum of square | Mean of square | F-value | Significant difference  (P value ˂0.05) |
| --- | --- | --- | --- | --- | --- | --- |
| Chlorophyll a | Salt Stress | 2 | 106875.25 | 53437.6 | 3834.41 | 0.000^***^ |
|  | Treatment | 3 | 24624.29 | 8208.09 | 588.97 | 0.000^***^ |
|  | Interaction  (Salt × treatment) | 3 | 2342.85 | 780.95 | 56.037 | 0.000^***^ |
| Chlorophyll b | Salt Stress | 2 | 62896.17 | 31448.08 | 2166.09 | 0.000^***^ |
|  | Treatment | 3 | 3589.78 | 1196.59 | 82.42 | 0.000^***^ |
|  | Interaction  (Salt × treatment) | 3 | 79.078 | 26.359 | 1.816 | 0.177 ns |
| Chl a/b | Salt Stress | 2 | 0.004 | 0.002 | 12.193 | 0.000^***^ |
|  | Treatment | 3 | 0.019 | 0.006 | 34.992 | 0.000^***^ |
|  | Interaction  (Salt × treatment) | 3 | 0.011 | 0.004 | 19.659 | 0.000^***^ |
| Total chlorophylls | Salt Stress | 2 | 319226.37 | 159613.18 | 4.298 | 0.028^**^ |
|  | Treatment | 3 | 51904.71 | 17301.57 | 0.466 | 0.709 ns |
|  | Interaction  (Salt × treatment) | 3 | 2092.69 | 697.566 | 0.019 | 0.996 ns |
| Carotenoids | Salt Stress | 2 | 1502.2 | 751.1 | 172.737 | 0.000^***^ |
|  | Treatment | 3 | 1087.77 | 362.59 | 83.386 | 0.000^***^ |
|  | Interaction  (Salt × treatment) | 3 | 101.181 | 33.727 | 7.756 | 0.001^**^ |
| N | Salt Stress | 2 | 554.644 | 277.322 | 2868.84 | 0.000^***^ |
|  | Treatment | 3 | 27.398 | 9.133 | 94.476 | 0.000^***^ |
|  | Interaction  (Salt × treatment) | 3 | 0.518 | 0.173 | 1.786 | 0.182 ns |
| P | Salt Stress | 2 | 1.664 | 0.832 | 76.308 | 0.000^***^ |
|  | Treatment | 3 | 0.179 | 0.060 | 5.475 | 0.007^**^ |
|  | Interaction  (Salt × treatment) | 3 | 0.022 | 0.007 | 0.667 | 0.582 ns |
| K | Salt Stress | 2 | 561.155 | 280.578 | 3117.53 | 0.000^***^ |
|  | Treatment | 3 | 45.775 | 15.258 | 169.535 | 0.000^***^ |
|  | Interaction  (Salt × treatment) | 3 | 0.655 | 0.218 | 2.424 | 0.096 ns |
| Na | Salt Stress | 2 | 312.642 | 156.321 | 2590.953 | 0.000^***^ |
|  | Treatment | 3 | 59.088 | 19.696 | 326.455 | 0.000^***^ |
|  | Interaction  (Salt × treatment) | 3 | 0.810 | 0.270 | 4.475 | 0.015^**^ |
| K/Na | Salt Stress | 2 | 4.031 | 2.016 | 895.882 | 0.000^***^ |
|  | Treatment | 3 | 0.477 | 0.159 | 70.659 | 0.000^***^ |
|  | Interaction  (Salt × treatment) | 3 | 0.051 | 0.017 | 7.602 | 0.001^**^ |
| Ca | Salt Stress | 2 | 417.825 | 208.913 | 2835.919 | 0.000^***^ |
|  | Treatment | 3 | 30.295 | 10.098 | 137.08 | 0.000^***^ |
|  | Interaction  (Salt × treatment) | 3 | 41.141 | 13.714 | 186.16 | 0.000^***^ |
| Parameters | Source | df | Sum of square | Mean of square | F-value | Significant difference  (P value ˂0.05) |
| Total soluble sugar | Salt Stress | 2 | 958.853 | 479.427 | 1559.957 | 0.000^***^ |
|  | Treatment | 3 | 42.755 | 14.252 | 46.372 | 0.000^***^ |
|  | Interaction  (Salt × treatment) | 3 | 19.663 | 6.554 | 21.327 | 0.000^***^ |
| Total soluble protein | Salt Stress | 2 | 5882.80 | 2941.40 | 4.808 | 0.014^**^ |
|  | Treatment | 3 | 8376.67 | 2792.22 | 4.564 | 0.020^*^ |
|  | Interaction  (Salt × treatment) | 3 | 8666.85 | 2888.95 | 4.722 | 0.012^**^ |
| Proline | Salt Stress | 2 | 3285.154 | 1642.577 | 1333.26 | 0.000^***^ |
|  | Treatment | 3 | 304.571 | 101.524 | 82.406 | 0.000^***^ |
|  | Interaction  (Salt × treatment) | 3 | 37.425 | 12.475 | 10.126 | 0.000^***^ |
| MDA | Salt Stress | 2 | 2.585 | 1.293 | 13.327 | 0.000^***^ |
|  | Treatment | 3 | 1.205 | 0.402 | 4.139 | 0.020^*^ |
|  | Interaction  (Salt × treatment) | 3 | 0.848 | 0.283 | 2.914 | 0.060 ns |
| H_2_O_2_ | Salt Stress | 2 | 3.683 | 1.842 | 16.742 | 0.001^**^ |
|  | Treatment | 3 | 2.855 | 0.952 | 8.652 | 0.000^***^ |
|  | Interaction  (Salt × treatment) | 3 | 1.195 | 0.398 | 3.621 | 0.031^*^ |
| POX | Salt Stress | 2 | 701.142 | 250.571 | 714.964 | 0.000^***^ |
|  | Treatment | 3 | 167.110 | 55.703 | 113.603 | 0.000^***^ |
|  | Interaction  (Salt × treatment) | 3 | 13.948 | 4.649 | 9.482 | 0.000^***^ |
| CAT | Salt Stress | 2 | 2404.455 | 1202.228 | 438.396 | 0.000^***^ |
|  | Treatment | 3 | 314.737 | 104.912 | 38.257 | 0.000^**8^ |
|  | Interaction  (Salt × treatment) | 3 | 3.768 | 1.256 | 0.458 | 0.715 ns |
| SOD | Salt Stress | 2 | 3019.483 | 1509.742 | 279.979 | 0.000^**8^ |
|  | Treatment | 3 | 281.728 | 93.909 | 17.415 | 0.000^***^ |
|  | Interaction  (Salt × treatment) | 3 | 6.355 | 2.118 | 0.393 | 0.759 ns |
| Phenolic | Salt Stress | 2 | 1119.142 | 559.571 | 608.009 | 0.000^***^ |
|  | Treatment | 3 | 664.515 | 221.505 | 240.679 | 0.000^***^ |
|  | Interaction  (Salt × treatment) | 3 | 5.381 | 1.794 | 1.949 | 0.154 ns |
| Fold change  SOS1 (root) | Salt Stress | 1 | 265.0 | 265.0 | 3459 | 0.0001^***^ |
|  | Treatment | 1 | 13.30 | 13.30 | 173.7 | 0.0001^***^ |
|  | Interaction  (Salt × treatment) | 1 | 0.2377 | 0.2377 | 3.103 | 0.1036ns |
| VHA2 (root) | Salt Stress | 1 | 131.0 | 131.0 | 4082 | 0.0001^***^ |
|  | Treatment | 1 | 12.20 | 12.20 | 380.0 | 0.0001^***^ |
|  | Interaction  (Salt × treatment) | 1 | 0.1620 | 0.1620 | 5.047 | 0.0443^*^ |
| VFK1 (root) | Salt Stress | 1 | 62.69 | 62.69 | 1851 | 0.0001^***^ |
|  | Treatment | 1 | 16.06 | 16.06 | 474.2 | 0.0001^***^ |
|  | Interaction  (Salt × treatment) | 1 | 0.01756 | 0.01756 | 0.5481 | 0.4853ns |
| KUP7(root) | Salt Stress | 1 | 98.01 | 98.01 | 2668 | 0.0001^***^ |
|  | Treatment | 1 | 16.40 | 16.40 | 446.5 | 0.0001^***^ |
|  | Interaction  (Salt × treatment) | 1 | 0.009025 | 0.009025 | 0.2457 | 0.6291ns |
| SOS1 (leaves) | Salt Stress | 1 | 80.15 | 80.15 | 3421 | 0.0001^***^ |
|  | Treatment | 1 | 15.46 | 15.46 | 6601 | 0.0001^***^ |
|  | Interaction  (Salt × treatment) | 1 | 4.275 | 4.275 | 182.5 | 0.0001^***^ |
| VHA2 (leaves) | Salt Stress | 1 | 35.25 | 35.25 | 2585 | 0.0001^***^ |
|  | Treatment | 1 | 8.688 | 8.688 | 637.0 | 0.0001^***^ |
|  | Interaction  (Salt × treatment) | 1 | 0.009506 | 0.009506 | 0.6970 | 0.4201ns |
| VFK1 (leaves) | Salt Stress | 1 | 195.2 | 195.2 | 2641 | 0.0001^***^ |
|  | Treatment | 1 | 33.87 | 33.87 | 458.3 | 0.0001^***^ |
|  | Interaction  (Salt × treatment) | 1 | 4.141 | 4.141 | 56.04 | 0.0001^***^ |
| KUP7 (leaves) | Salt Stress | 1 | 231.1 | 231.1 | 1194 | 0.0001^***^ |
|  | Treatment | 1 | 20.82 | 20.82 | 107.6 | 0.0001^***^ |
|  | Interaction  (Salt × treatment) | 1 | 0.3053 | 0.3053 | 1.577 | 0.2330ns |

Two-Way analysis of variance (ANOVA) showing effects of nanoparticles (NPs), bulk fertilizers and salt, and their interactions, on growth and various physiological parameters of faba bean plant. The significant difference was represented as *, **, *** = significant at P value ˂ 0.05, 0.01, and 0.001 levels, respectively. ns = non-significant

In a two-way ANOVA, "df" refers to degrees of freedom. Degrees of freedom represent the number of independent pieces of information available to estimate a parameter or calculate a statistic. In the context of ANOVA, they are crucial for determining the critical F-values and p-values used to assess the statistical significance of main effects and interaction effects.
